# Light Scattering Contrast Inversion of Single Metal Nanoparticles Inside a Nanofluidic Channel

**DOI:** 10.1021/acs.jpcc.6c03991

**Published:** 2026-07-28

**Authors:** Lova Wilske, Joachim Fritzsche, Barbora Špačková, Bohdan Yeroshenko, Christoph Langhammer

**Affiliations:** † Department of Physics and Astronomy, Chalmers University of Technology, SE-412 96 Göteborg, Sweden; ‡ FZU - Institute of Physics of the Czech Academy of Sciences, 182 00 Prague 8, Czech Republic

## Abstract

Nanofluidics enables the high-precision control of fluid flow, as well as of tiny particles and molecules, at the nanoscale. Combined with optical microscopy and spectroscopy, this has generated an experimental platform for the study of single nanoentities by means of light, often enhanced by nanoscale interference effects. However, in the realm of metal nanoparticles inside nanofluidic systems, contradictory light scattering properties have been reported, and the underlying physics is poorly understood. Here, we systematically investigate Pt and Au nanodisks nanofabricated into nanotrenches in a poly­(methyl methacrylate) matrix to emulate nanofluidic systems and find that their light scattering signature can appear both brighter and darker than the trench alone, or become completely invisible, depending on the specific interplay between trench and nanodisk diameter. An analytical model based on the electrostatic approximation allows us to qualitatively understand the fundamental physics of this effect based on the interference of light scattered by a metal nanodisk and a nanofluidic structure. To corroborate the model and demonstrate an application in a fully functional nanofluidic system, we show that the optical appearance of single metal nanodisks inside a fully enclosed nanofluidic channel inside an SiO_2_ matrix can be dynamically tuned from dark to bright to completely invisible by adjusting the refractive index of a liquid inside the channel. We predict that this effect will find application in optical sensing in nanofluidic systems for volumes smaller than the diffraction limit of light.

## Graphical abstract

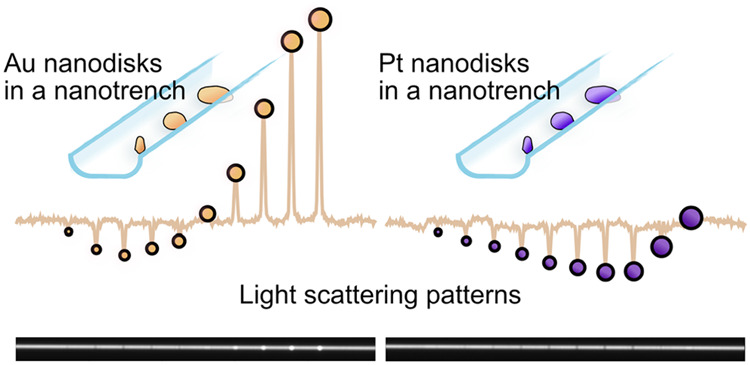

## Introduction

Nanofluidic systems enable precise control of fluid flow at the nanoscale and thus hold great promise as experimental platform for applications in biology, chemistry, and material science.^1–4^ Furthermore, the prospect of isolating tiny entities, such as molecules or nanoparticles, inside such nanofluidic systems for observation, and the possibility to, with high precision, manipulate the local chemical environment around these entities, makes nanofluidic channels highly relevant for studying individual molecules and nanoparticles. To realize such studies, numerous experimental methods have been developed, including mass spectroscopy,^5^ resistivity measurements,^6,7^ chromatography,^8^ mass flowmetry,^9^ and different optical microscopy^10–17^ and spectroscopy^18–20^ techniques. Among these methods, optical microscopy stands out as noninvasive and low-cost candidate that provides high spatial and temporal resolution. However, as a general challenge in this field, all characterization methods listed above, including specifically also optical microscopy, often need to operate close to their limit of detection, due to the inherently tiny signals generated by single particles and molecules. To push the detection limit of optical microscopy for nanofluidic applications in particular, strategies like fluorescent probes^10–12^ or plasmonic sensing^19,21,22^ have been successfully demonstrated. More recently, we have introduced nanofluidic scattering microscopy^17^ (NSM) and spectroscopy^20^ (NSS) that enhance the light scattering-based optical contrast of single molecules and nanoparticles based on the principle of optical *interference*. Specifically, NSM uses the dark-field illumination of a nanofluidic channel, and the collection of the light scattered from said channel. If a nanoparticle is localized inside the nanochannel the total intensity of the scattered light can be attributed to a contribution from the channel, *I*_channel_, a contribution from the particle, *I*_particle_, and the contribution from the interference of light scattered from the channel and the particle, *I*_interference_, and thus be written as

1 $$I_{\mathrm{tot}}=I_{\mathrm{channel}}+I_{\mathrm{particle}}+I_{\mathrm{interference}}$$

Here, the fact that $I_{int\mathrm{erf}erence}\propto \sqrt{I_{channel}I_{particle}^{\prime }}$ , where *I*_particle_^′^ is the intensity associated with the real part of the particle refractive index, is the key to the signal enhancement.^17^ Specifically, by differential imaging, i.e., the subtraction of an image of a nanochannel without particle inside (*I*_channel_) from an image of an identical nanochannel with particle inside, nanosized objects can be resolved even if *I*_particle_ is below the limit of detection, provided the contribution of *I*_Interference_ is significant.

Based on this principle, we have introduced NSM to determine the molecular weight and hydrodynamic radius of single biomolecules and biological nanoparticles like extracellular vesicles.^17,23^ Furthermore, we have applied both NSM and NSS to study catalytic reactions over single catalyst nanoparticles, by quantitatively analyzing the refractive index change induced by the reaction in the reactant solution inside the nanofluidic channel that hosts the catalyst nanoparticle.^24,25^ However, the NSM/NSS concepts have so far not been applied to metal nanoparticles, despite the potential of enabling their efficient detection, tracking, and characterization, which is of particular interest for colloidal nanocrystal synthesis, plasmonics and catalysis.

To this end, the physics of light interacting with a metal nanoparticle placed *inside* another nanosized – and therefore diffraction limited – object that itself also scatters light, such as a nanofluidic channel used for NSM/NSS, is not well understood. This becomes obvious when comparing the few studies available, which report that metal particles can appear either bright or dark in comparison to a nanochannel and that the magnitude of the scattering intensity difference between nanochannel and metal nanoparticle (the “scattering contrast”) can vary greatly between platforms, depending on the particle size and composition, the nanofluidic system’s dimensions, and the microscope configuration.^13,14,24,26,27^ Understanding the origin of these, at first contradictive, observations is fundamentally interesting and of practical importance because a large scattering contrast – bright or dark – is key in any application that targets the tracking and study of metal nanoparticles in nanofluidic systems using optical microscopy and spectroscopy.

In this work, we therefore investigate the physics of the interaction of light with metal nanoparticles localized inside nanofluidic channels embedded in dielectric matrix transparent to visible light, to understand the origin of the reported contradictory optical signatures of such systems. We do this first experimentally by nanofabricating a model system that emulates fully enclosed and functional nanofluidic channels by open “nanotrenches”, electron-beam lithography (EBL) fabricated into a PMMA layer spin coated onto a thermally oxidized Si wafer substrate. We use this model system because it: (i) enables the Scanning Electron Microscopy (SEM) based size characterization of linear arrays of Au and Pt nanodisks with systematically varied sizes and 5 μm interparticle-distance that we EBL-fabricated into the nanotrenches since it is “open”; (ii) ensures close similarity to fully enclosed and functional nanofluidic systems in terms optical properties since both of them constitute diffraction-limited 1D structures embedded in a dielectric matrix; (iii) facilitates the experimental assessment of the light scattering properties using reflection-mode dark-field scattering microscopy. Subsequently, to qualitatively analyze the found experimental trends for the scattering contrast of nanodisks with systematically varied sizes inside nanofluidic trenches, we develop an analytical model for the wavelength dependent scattering cross section of a homogeneous cylinder embedded in a dielectric matrix and decorated with a metal nanoparticle modeled as a perfect spheroid. Finally, to corroborate the validity of the model, and demonstrate a first application on a *fully enclosed* and thus functional nanofluidic system, we study the change in optical scattering contrast of differently sized Pt nanodisks as a function of the refractive index (RI) of the liquid inside a single nanofluidic channel. In this way, we can demonstrate that the optical appearance of single metal nanoparticles can be dynamically tuned from dark to bright or even completely invisible. We predict that this effect will find application in optical sensing in nanofluidic systems from volumes smaller than the diffraction limit of light.

## Methods

### Nanofabrication

The two kinds of samples used in this work, i.e., nanotrenches and fully integrated nanofluidic chips, were fabricated in the ISO class 6 cleanroom facilities MC2 at Chalmers. Nanotrenches: the sample was fabricated from a 4-in. (100) silicon wafer with 191 nm thick dry oxide in three steps. In the first step, 9 linear Au nanodisk arrays (8 of them to be placed in trenches) were fabricated by patterning an MMA/PMMA resist film by EBL (JEOL JBX 9300FS, dose 2000 μC/cm^2^) and subsequently depositing a 5 nm Cr adhesion layer followed by a layer of 60 nm Au, using electron beam evaporation (Lesker PVD 225). The resist mask was then dissolved in MICROPOSIT Remover 1165, thereby lifting off the metal layer except in the patterned regions. As the second step, the process was repeated to fabricate identical Pt nanodisk arrays, this time e-beam evaporating 5 nm Cr and 60 nm Pt. To ensure the correct positioning of the Pt disk arrays with respect to the Au ones, we used alignment marks fabricated with EBL during the previous exposure. In the final step, we spin-coated the wafer with a 140 nm thick PMMA layer (950k-A3) and EBL-patterned nanotrenches along the 16 (8 Au and 8 Pt) nanodisk arrays, again using the previously fabricated alignment marks. In addition to the trenches, a larger area (220 × 220 μm^2^) was patterned at the position of the ninth Au and Pt particle arrays to enable studies of single disks on an open surface without interference from a trench. Nanofluidic chip: The fabrication process of fluidic chips has previously been described in detail by Levin et al.^12^ and Špačková et al.^17^ and is here summarized as follows: We started from a 4-in., 1 mm thick, (100) silicon wafer and cleaned it with Standard Clean 1, HF bath, and Standard Clean 2, before a 2100 nm wet oxide was thermally grown on the wafer surface. A set of 16 alignment marks were subsequently fabricated with EBL onto each of the 52 identical chips on the wafer. Next, nanochannels with 192 nm width were patterned through a film of resist (950k-PMMA A4, 60 s, 4000 rpm spin coating, plus 5 min baking at 180 °C) using EBL (dose 1600 μC/cm^2^), and subsequently etched into the thermal oxide to a depth of 70 nm using reactive ion etching (Oxford Plasma lab 100 ICP180) in CHF_3_ plasma. The sample was then cleaned in a 130 °C H_2_SO_4_:H_2_O_2_ solution. Linear Au and Pt nanodisk arrays with disk diameters ranging from 10 to 100 nm in 13 steps were thereafter EBL fabricated into the nanochannels in two steps using the same procedure as described for the nanotrench samples. The disk arrays featured nominal particle heights of 5 nm Cr plus 40 nm Au, and 5 nm Cr plus 40 nm Pt, respectively. In the next step, microfluidic channels with 50 × 1.5 μm^2^ dimensions and inlet holes that connect the chip to the sample holder, necessary for introducing liquids to the nanofluidic regions, were patterned with a laser writer (MA6, Suss MicroTec) and etched with a CF_4_ plasma (Advanced vacuum Batchtop m/91) and deep reactive ion etching (Plasma Pro 100, Oxford Instruments) respectively. The wafer with the patterned structures was then cleaned in acetone. Subsequently, a 4-in. Borofloat 33 glass wafer to be used as the lid to hermetically seal the fluidic structures, was cleaned in a 130 °C H_2_SO_4_:H_2_O_2_ solution. After cleaning, the surfaces of the nanopatterned Si wafer and the Borofloat 33 glass wafer were activated in oxygen plasma (1 min, 100 W, 250 mTorr) to enable prebonding of the two wafers by pressing them together. Finally, after the prebonding, the two wafers were fusion-bonded at 560 °C in an inert N_2_ atmosphere for 5 h. As a final step, the bonded wafer was diced into 52 10 by 10 mm^2^ identical chips containing the designed fluidic system (Disco DAD3350).

### Instrumentation

Dark-field scattering microscopy measurements were performed on an upright dark-field microscope (Nikon Eclipse LV150N) equipped with a Nikon 50x ELWD dark-field objective and a white LED light source (Thorlabs Solis-3C). For measurements of the PMMA nanotrench samples (Figure 1, 2, and 4), narrow band filters (Thorlabs FBH500–10, FBH600–10, and FBH700–10) were added in the collection pathway. Image acquisition was conducted with a sCMOS camera (Andor Zyla 4.2 sCMOS), with an effective 10 s exposure time. All images were subsequently normalized by a bright-field image of a Ag mirror, acquired using the three narrow band filters in turn.

The dark-field scattering spectra of an empty nanotrench and of the single nanodisks displayed in Figure 2 were collected by inserting a liquid crystal tunable bandpass filter (Kurios K2WL1) is the collection pathway of the microscope. Dark-field images were then collected for 52 different wavelengths ranging between 450 and 705 nm in steps of 5 nm. The effective exposure time for each image in the trench spectrum was 3 s and for the nanodisks it was 10 s. All images were subsequently normalized by a bright-field image of a Ag mirror, acquired at the corresponding wavelength. From each imaged nanodisk a wavelength dependent intensity (spectrum) was extracted by summing the intensity counts of the pixels of each nanodisk (11 by 11 pixels, that correspond to a 1.45 by 1.45 μm^2^ area). In addition, the average intensity from the substrate (background) was subtracted. For the nanotrench spectrum in Figure 2a, 11 pixels along the trench and 25 pixels across the trench, were summed in the analysis.

For the experiments with the nanofluidic chip presented in Figure 6, we attached a 3D-printed aperture around the objective. The aperture blocks all light illuminating the sample through the ring illumination objective except through an 83 mm gap on each side of the aperture (each gap correspond to ∼25 ° angle of the illumination ring). In this way, the nanochannels are illuminated from the side while the microchannels are illuminated in a direction parallel to them, which effectively suppresses light scattered by the microchannels. A sCMOS camera (Andor Zyla 4.2 sCMOS) operated with 0.15 s exposure time was used for image acquisition. A pressure controller (Fluigent MFCS-EX) was used to control the liquid flow through the nanofluidic chip. Four liquids were used in the experiments: Milli-Q IQ 7000 water and ethylene glycol (99.5%, from Acros Organics), ethanol (95%, from Solveco) and trans-Cinnamaldehyde (99%, Thermo Scientific Chemicals).

For sample characterization we used Zeiss Supra 60 VP SEM at 7 kV acceleration voltage and 3.3–3.7 mm working distance. Prior to SEM analysis a 1 nm Au layer was sputtered onto the nanotrench PMMA sample to protect the chip from beam damage by making it more conductive.

## Results and Discussion

To systematically investigate the interplay between light scattered from a nanofluidic system, and light scattered and absorbed by a plasmonic metal nanoparticle inside it, we electron-beam-lithography-fabricated (EBL) nanotrenches with a constant 200 nm width by etching them into a 140 nm thick poly­(methyl methacrylate) (PMMA) film spin coated onto a Si wafer with 191 nm thermal oxide (see Methods for details). With nanotrench, we mean here a trench-like nanostructure etched into PMMA that is not sealed with a lid and thus constitutes an “open nanofluidic channel”. This system on one hand allows us to image the nanoparticles inside using electron microscopy and on the other hand optically behaves very similarly to a completely enclosed nanochannel, which is an efficient scatterer of visible light if its cross-sectional diameter is on the order of tens to few hundred nanometers.^17^ Within these trenches, we nanofabricated linear arrays of Au and Pt nanodisks, respectively, with 5 μm interparticle separation (Figure 1a–d). Along the array, the disk diameter was increased from ∼30 nm to ∼120 nm in nine steps (see SI Figure S1 for SEM images). The deposited metal thickness (disk height) was kept constant at 60 nm. Subsequently, we illuminated the trench with the nanodisk linear array through a ring-illumination dark-field objective on an upright optical microscope in reflection mode (see Methods for details about the setup) using a white light LED source. To select a specific illumination wavelength, we placed bandpass filters transmitting 500 ± 10 nm, 600 ± 10 nm and 700 ± 10 nm, respectively, in the light collection pathway (Figure 1e–j).

Starting at 500 ± 10 nm scattered light wavelength and with the Au system, we find that all but the largest nanodisks appear as dark diffraction limited spots compared to the bright nanotrench in a dark-field scattering image (inset in Figure 1e). The third-largest 104 nm nanodisk is hardly visible at all, and the two largest ones (108 and 120 nm) appear as bright diffraction limited spots that scatter more light than the trench. This becomes even clearer when plotting the scattering intensity trace obtained along the trench with the nanodisks relative to the scattering intensity trace of an identical empty nanotrench (*I*_relative_ = *I*_*t*rench+particle_ – *I*_trench_), as depicted in Figure 1e. Clearly, the small Au disks appear as negative peaks, the 104 nm particle is essentially invisible, and the 108 and 120 nm disks appear as distinct positive peaks. Interestingly, similar analysis of the Pt nanodisk array reveals that all particles across the board appear as distinct dark diffraction limited spots and thus scatter less light than the trench alone (Figure 1f).

Collecting instead 600 nm ± 10 nm scattered light from the sample reveals that for the Au system, the contrast between the optically darker (than the trench) small particles and the brighter large ones has increased, as well as that the disk diameter at which the inversion from dark to bright occurs is shifted from 104 nm to between 72 and 80 nm (Figure 1g). For the Pt nanodisk system, the relative negative (=darker) scattering intensity contrast with respect to the trench alone is larger and the largest disk (110 nm) is essentially invisible or just slightly brighter than the trench (Figure 1h). Finally, analyzing light scattered from the sample at 700 nm ± 10 nm wavelength reveals a return to a very similar response of both Au and Pt systems as we observed for 500 nm ± 10 nm (Figure 1i,j).

**Figure 1:**
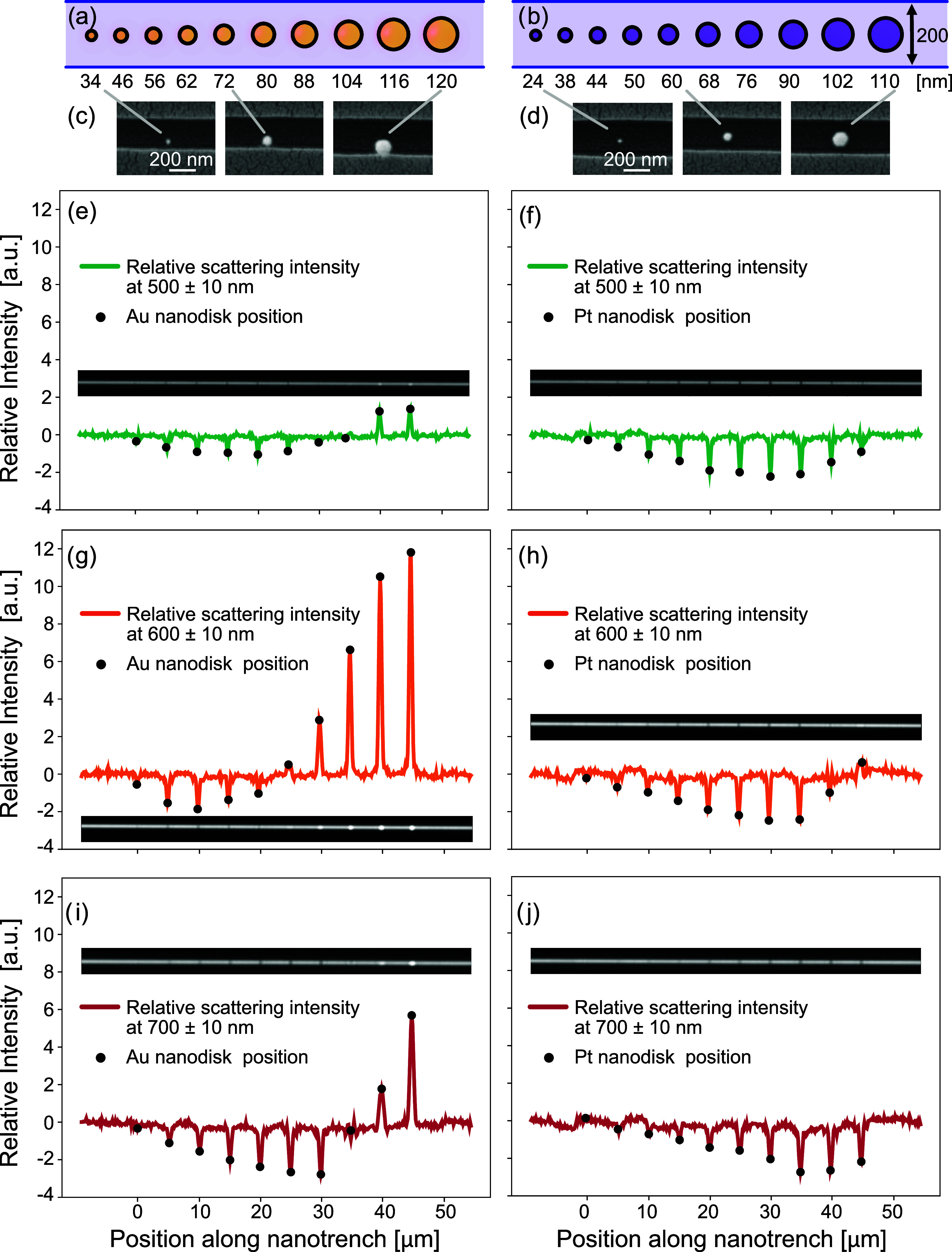
(a, b) Schematic top-view representations of the size gradient Au or Pt nanodisk array nanofabricated into a PMMA nanotrench. Nanodisk diameters and trench width are indicated. The internanodisk distance is 5 μm. (c) Selected SEM images of Au nanodisks inside a PMMA nanotrench. (d) Selected SEM images of Pt nanodisks inside a PMMA nanotrench. (e) Light scattering intensity of the Au nanodisk array measured for a 200 nm wide nanotrench at 500 ± 10 nm irradiated wavelength plotted relative to the scattering intensity of the nanotrench at a position without particle. A positive peak thus indicates a nanodisk appearing brighter than the trench and a negative peak a nanodisk appearing darker than the trench. The inset depicts an absolute dark-field scattering intensity image of the nanotrench with the Au nanodisk array inside that is normalized by a bright field reference. (f) Same as (e) but for the Pt nanodisk array. (g) Same as (e) but for 600 nm ± 10 nm scattered light. (h) Same as (f) but for 600 nm ± 10 nm scattered light. (i) Same as (e) but for 700 nm ± 10 nm scattered light. (j) Same as (f) but for 700 nm ± 10 nm scattered light.

To further discuss these initial results for Au and Pt nanodisks of different diameters in a PMMA nanotrench, it is relevant to characterize the light scattering properties of the trench and the disks alone. The corresponding spectrum of a 200 nm wide empty nanotrench is depicted in Figure 2a. In order to acquire similar optical spectra of the nanodisks alone, we EBL-nanofabricated a separate sample with Au and Pt nanodisks placed on an open oxidized Si wafer surface. The sample comprised individual Au and Pt nanodisks arranged in arrays with disk diameter ranging from ∼38 nm to ∼135 nm and a constant thickness of 60 nm (SEM images are presented in SI Figure S3). Corresponding single particle dark-field scattering spectra for the Au (Figure 2b) and Pt (Figure 2c) systems reveal distinct plasmonic resonances. In agreement with the literature, the LSPR peaks shift to longer wavelengths for increasing diameter for both metals. Furthermore, the Pt resonances are blue-shifted compared to Au and broader because they are more damped in the lossy Pt system due to coupling to inter- and intraband transitions, which also favors absorption over scattering.^28–30^

**Figure 2:**
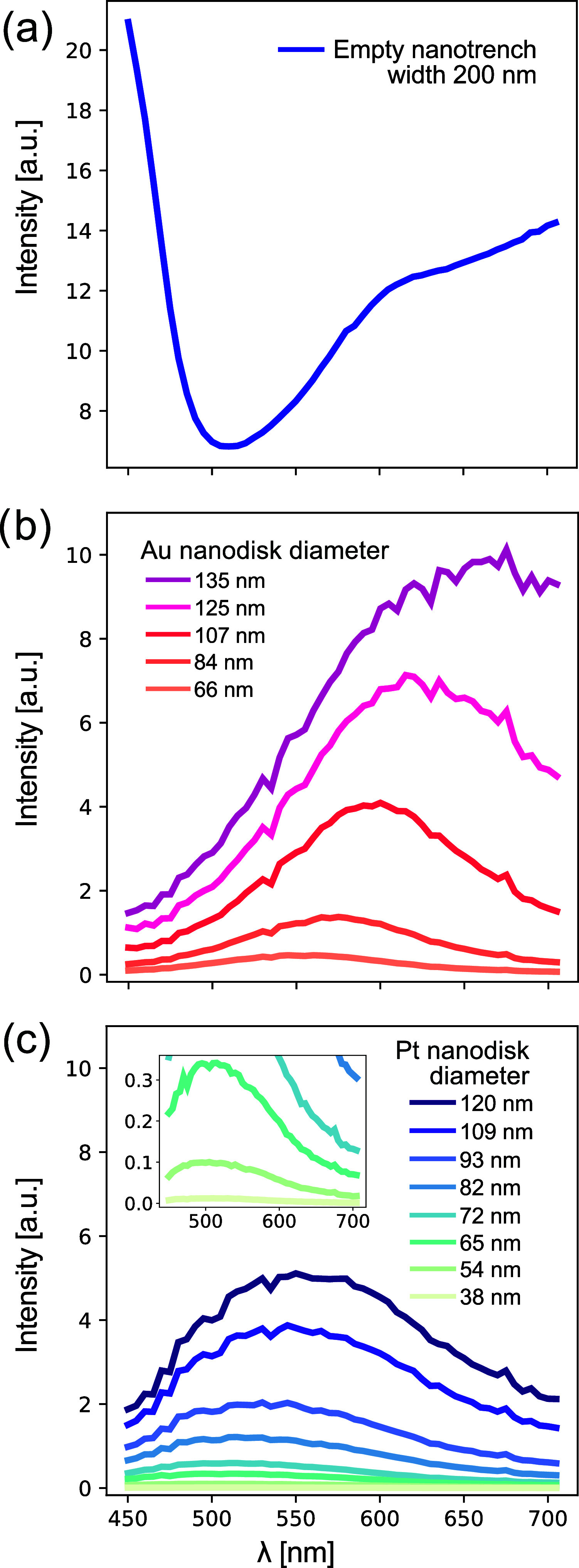
(a) Dark-field scattering spectrum of an empty nanotrench (200 nm wide and 140 nm deep). (b) Dark-field scattering spectra of single Au nanodisks with different diameters and constant nominal 60 nm evaporated metal thickness, nanofabricated onto an oxidized Si wafer of the same type as used for the nanotrench samples. (c) Dark-field scattering spectra of single Pt nanodisks, fabricated in the same manner as in (b). In the inset, the intensity scale of the spectra is adjusted to display the spectra of the 3 smallest nanodisks in greater detail.

To understand the physics of the observed effect, i.e., that a plasmonic nanoparticle localized inside a nanotrench can appear both brighter and darker than the trench alone depending on its size, we develop an analytical model with the ambition to *qualitatively* capture the experimental trends (i.e., we do not attempt to reproduce the experimental values exactly). For this purpose, we first derive an analytical expression for the scattering cross section, σ_*s*,*c*_, of an infinitely long cylinder of radius *a* normally irradiated by unpolarized light using Mie theory in the Rayleigh limit (SI Figure S4–S5a, see SI Section 2 for derivation). To do so, we combine the contributions of TE and TM polarizations by multiplying the respective scattering efficiencies, *Q*_s,TM_; *Q*_s,TE_, with the physical cross section of the cylinder, 2*al*, along an arbitrary length, *l*, along which the scattering is to be evaluated. We obtain

2 $$\sigma_{s,c}=2\mathrm{al}\frac{1}{2}\left(Q_{s,\mathrm{TM}}+Q_{s,\mathrm{TE}}\right)=\frac{A^{2}k^{3}l}{4}{\left(m^{2}-1\right)}^{2}\left(\frac{1}{2}+\frac{1}{{\left(m^{2}+1\right)}^{2}}\right)$$

where the wavenumber *k* = 2π*n*_o_/λ (with λ corresponding to the wavelength and *n*_o_ to the RI of the medium the cylinder is embedded in), *A* = π*a* ^2^ and *m* = *n*_c_/*n*_o_ the relative RI defined as the ratio between the RI inside the cylinder, *n*_c_, and the RI outside, *n*_o_. For orthogonal illumination of a cylinder the scattering efficiency is larger for TM polarization than TE, see SI Figure S5b.

Subsequently, we approximate the metal particles as spheroids in the so-called Modified Long Wavelength Approximation (MLWA) in the electrostatic regime to account for dynamic depolarization and radiation damping effects in their interaction with visible light,^31,32^ and we only consider dipolar plasmonic modes. This yields an expression for their scattering cross section, σ_s,p_, as

3 $$\sigma_{s,p}=\frac{k^{4}}{6\pi }{\left|\alpha_{p}\right|}^{2}$$

where

4 $$\alpha_{p}=\frac{\alpha }{1-i\frac{k^{3}}{6\pi }\alpha -\frac{k^{2}}{2\pi d}\alpha }$$

and where

5 $$\alpha =V_{p}\frac{n_{p}^{2}-n^{2}}{n^{2}+L\left(n_{p}^{2}-n^{2}\right)}$$

with *V*_p_ being the particle volume, *n*_p_ its complex RI, and *n* the effective RI of the environment (widely approximated as *n* = (*n*_SiO2_ + *n*_Air_)/2 for glass-supported nanoparticles, as the case also here in our work) and *L* a geometrical factor (see SI Section 2 for complete derivation).

Equipped with the above analytical descriptions of the two independent systems, we now set out to develop an analytical expression for the scattering cross section of their combination, i.e., a metal nanoparticle inside a cylinder embedded in a dielectric medium, by treating the particle and a *fraction* of the cylinder occupied by the particle as an ideal dipole with an effective polarizability that can be written as

6 $$\alpha_{\mathrm{tot}}=\alpha_{c}+\alpha_{p}$$

where α_c_ is the polarizability of a cylinder with length *l*. To extract α_c_, we rearrange eq 2 to

7 $$\sigma_{s,c}=\frac{k^{4}}{6\pi }{\left|\sqrt{\frac{3\pi }{2}}\frac{A\sqrt{l}}{\sqrt{k}}\left(m^{2}-1\right)\sqrt{\frac{1}{2}+\frac{1}{{\left(m^{2}+1\right)}^{2}}}\right|}^{2}=\frac{k^{4}}{6\pi }{\left|\alpha_{c}\right|}^{2}$$

Subsequently, we note that the particle occupies a finite section of the cylinder, which in the far field corresponds to the (wavelength-dependent) diffraction limited spot. Accordingly, as the key assumption of the model, we define that the length, *l*, of the cylinder along which we treat the combined cylinder - particle system as an ideal dipole corresponds to the size of the diffraction limited spot at a given wavelength. This means that by substituting *k* = *l* ^–1^ the expression for α_c_ in eq 6, reads as

8 $$\alpha_{c}=\sqrt{\frac{3\pi }{2}}\mathrm{Al}\left(m^{2}-1\right)\sqrt{\frac{1}{2}+\frac{1}{{\left(m^{2}+1\right)}^{2}}}$$

The total scattering cross section within a diffraction limited spot encapsulating a particle located in a cylinder observed over a length *l* = λ/2π*n*_o_, then becomes

9 $$\sigma_{s,\mathrm{tot}}=\frac{k^{4}}{6\pi }{\left|\alpha_{c}+\alpha_{p}\right|}^{2}$$

This expression can be expanded into the sum of the scattering cross sections of the cylinder, σ_s,c_, and the particle, σ_s,p_, respectively, plus a third term that represents the interference between the two, σ_s,int_

10 $$\sigma_{s,\mathrm{tot}}=\sigma_{s,c}+\sigma_{s,p}+2\mathrm{sign}\left(\alpha_{c}\alpha_{p}^{\prime }\right)\sqrt{\sigma_{s,c}\sigma_{s,p}^{\prime }}$$

where α_p_^′^ is real part of the particle polarizability and σ_s,p_^′^ is the contribution to the scattering cross section corresponding to the real part of the particle polarizability, $\sigma_{s,p}^{\prime }=\frac{k^{4}}{6\pi }{\left|\alpha_{p}^{\prime }\right|}^{2}$ . For a complete derivation, see SI Section 2.

We can now apply this formalism to calculate the total scattering cross section spectrum of spheroidal Au particles positioned in an air-filled 200 nm wide cylinder embedded in a SiO_2_ matrix (Figure 3a) – or to be more precise, we calculate the spectrum of a diffraction limited spot, centered at the position of a metal particle localized inside a cylinder. We here set SiO_2_ to be the embedding medium, even though the RI of PMMA and SiO_2_ are slightly different (*n*_PMMA_ = 1.49, and *n*_SiO_2__ = 1.46), as using SiO_2_ corresponds more accurately to a fully enclosed nanofluidic system that we discuss in the last part of this work. In the spectrum, a distinct peak can be noticed for the system with particles larger than 80 nm. This peak is blue-shifted compared to the LSPR peak of the corresponding particles modeled when localized on an open surface (SI Figure S6). This peak shift can be attributed to the interference term contribution (cf. eq 10) to the combined particle-cylinder-system’s scattering cross section (Figure 3b), which contributes an increase to the scattering cross section in the blue part of the spectrum and a decrease for longer wavelengths. Furthermore, we note that the sign of this interference contribution depends on the real part of the polarizability of the cylinder and the particle. While a cylinder filled with air, and embedded in SiO_2_, has a strictly negative real-valued polarizability, the real part of the polarizability of an Au spheroidal particle can vary between positive and negative, thus altering the sign of the interference term in eq 10 (see SI Figure S6 for modeled spectrum of the particle polarizability). The change of sign in α_p_^′^ is a consequence of the relation between the complex RI of Au and the RI of the surrounding medium.

**Figure 3:**
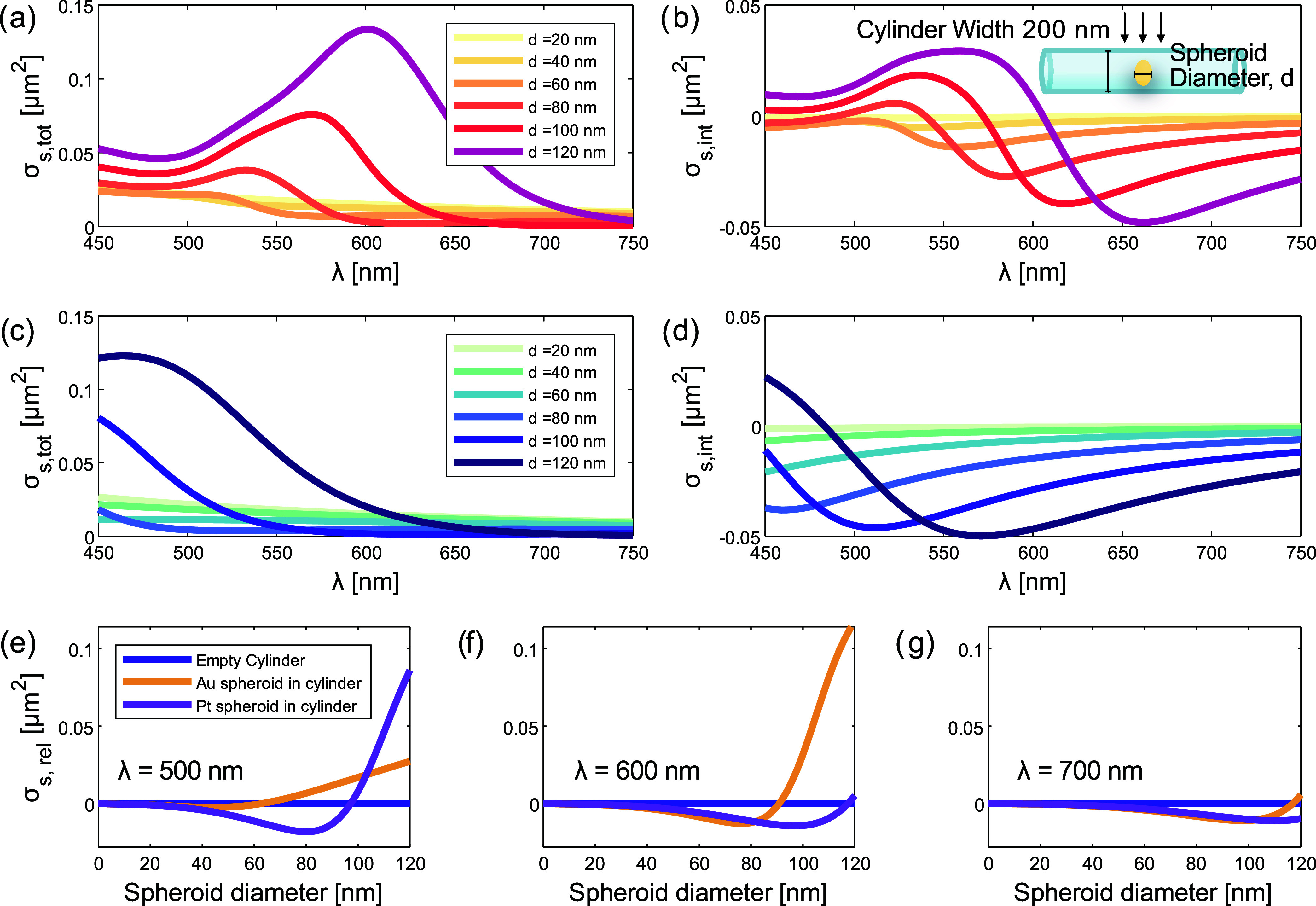
(a) Total scattering cross section spectrum, σ_s,tot_, calculated using eq 10 for spheroidal Au nanoparticles with a constant 60 nm height and varying circular diameter, located in an air-filled cylinder with 200 nm diameter that is embedded in a SiO_2_ matrix. The particle-cylinder system is illuminated at normal incidence to the cylinder long axis with unpolarized light, modeled as a combination of the contributions from TE and TM polarizations. (b) Calculated interference term cross section spectrum (eq 10), σ_s,int_, for the Au spheroid – cylinder system representing the wavelength-dependent contribution to the total scattering cross section of the system. (c) Same as (a) but for Pt spheroids. We note the blue-shift of the spectral features compared to Au as the consequence of the intrinsically more blue-shifted LSPR in Pt spheroids. (d) Same as (b) but for Pt spheroids. (e) Calculated 500 nm wavelength scattering cross sections of Au and Pt spheroids with different diameters inside a cylinder with 200 nm diameter relative to the scattering cross section of the empty air-filled cylinder, σ_s,rel_,. Evidently, the interference term may lead to both increased or decreased scattering from the position of the particle inside the cylinder, depending on the diameter of the spheroid. This qualitatively reproduces well the corresponding experimental observations summarized in Figure 1. (f) same as (e) but for 600 nm wavelength. (g) same as (e) but for 700 nm wavelength.

Similarly, the total scattering cross section spectrum of the Pt spheroidal particle-cylinder system and the interference contribution to it can be analyzed using our model. We find a qualitatively similar behavior with the main difference being a distinct blue-shift of the LSPR-related features (Figure 3c,d). This is the consequence of the intrinsically blue-shifted LSPR of the Pt spheroids compared to the Au system (SI Figure S6).

To, as the next step, qualitatively relate the predictions by our model to the experimental observations presented above, we computed the relative scattering cross sections, σ_s,rel_ = σ_s,tot_ – σ_s,c_, at 500 nm (Figure 3e), 600 nm (Figure 3f), and 700 nm (Figure 3g) for Au and Pt spheroids with different diameters and at constant 60 nm height inside a cylinder of 200 nm diameter embedded in SiO_2_. The obtained σ_s,rel_ – spheroid diameter relationships at the three wavelengths are indeed qualitatively similar to the corresponding experimental results depicted in Figure 1. Most importantly, for 600 nm wavelength the model predicts that Au spheroids larger than 90 nm scatter more light than the cylinder alone and spheroids smaller than 90 nm scatter less, whereas all Pt spheroids are darker than the cylinder alone (Figure 3f). Our model thus clearly captures the key experimental observation, namely that a scattering contrast *inversion* at the position of a plasmonic metal nanoparticle localized inside a nanotrench can occur for a given combination of nanoparticle dimension and permittivity, and light wavelength. It also confirms that for a specific combination of these parameters a plasmonic particle can become completely invisible. As the mechanism, we identify the interference between light scattered by the cylinder/trench and the metal nanoparticle, as evident from eq 10.

Returning to our experiments, so far, we have only considered a scenario where we have kept the dimensions of the nanotrench constant and varied the dimensions and material of the nanodisks inside it. However, from the derived analytical formalism, it becomes clear that the cross-section area of the trench (or the radius, *a*, of the cylinder) also is expected to sizably contribute to σ_s,tot_. It is therefore interesting to experimentally implement nanotrenches with different width and again decorate them with arrays of Au and Pt particles of systematically varied diameter that again range from ∼30 nm to ∼120 nm, and to analyze their scattering response at 600 ± 10 nm (Figure 4a–g, and SI Figure S7, S9), 500 ± 10 nm (SI Figure S8), and 700 ± 10 nm (SI Figure S10). We observe that the relative scattering intensity of the disks in the trench relative to the trench alone again can vary between negative and positive as the disk diameter is increased. It is also evident that the particle diameter at which the transition from negative to positive occurs, i.e., the *scattering contrast inversion point*, depends on the width of the trench for both the Pt and Au systems. Specifically, for a narrower trench this scattering contrast inversion occurs at a smaller disk diameter than for a wider trench. By interpolating the diameter of the first disk with dark and last disk with bright scattering contrast along the nanotrench, we can estimate the disk diameter at which the inversion occurs and where a nanodisk will be invisible. Accordingly, we extracted the inversion point disk diameter for 7 trenches with different widths for the Au system and for 5 trenches with different widths for the Pt system (Figure 4h - see SI Figure S2, and SI Section 3 for details). This analysis reveals a strictly positive trend for both metals where the diameter of the “invisible” Au nanodisk always is smaller than the corresponding Pt one when located in a nanotrench of the same width.

**Figure 4:**
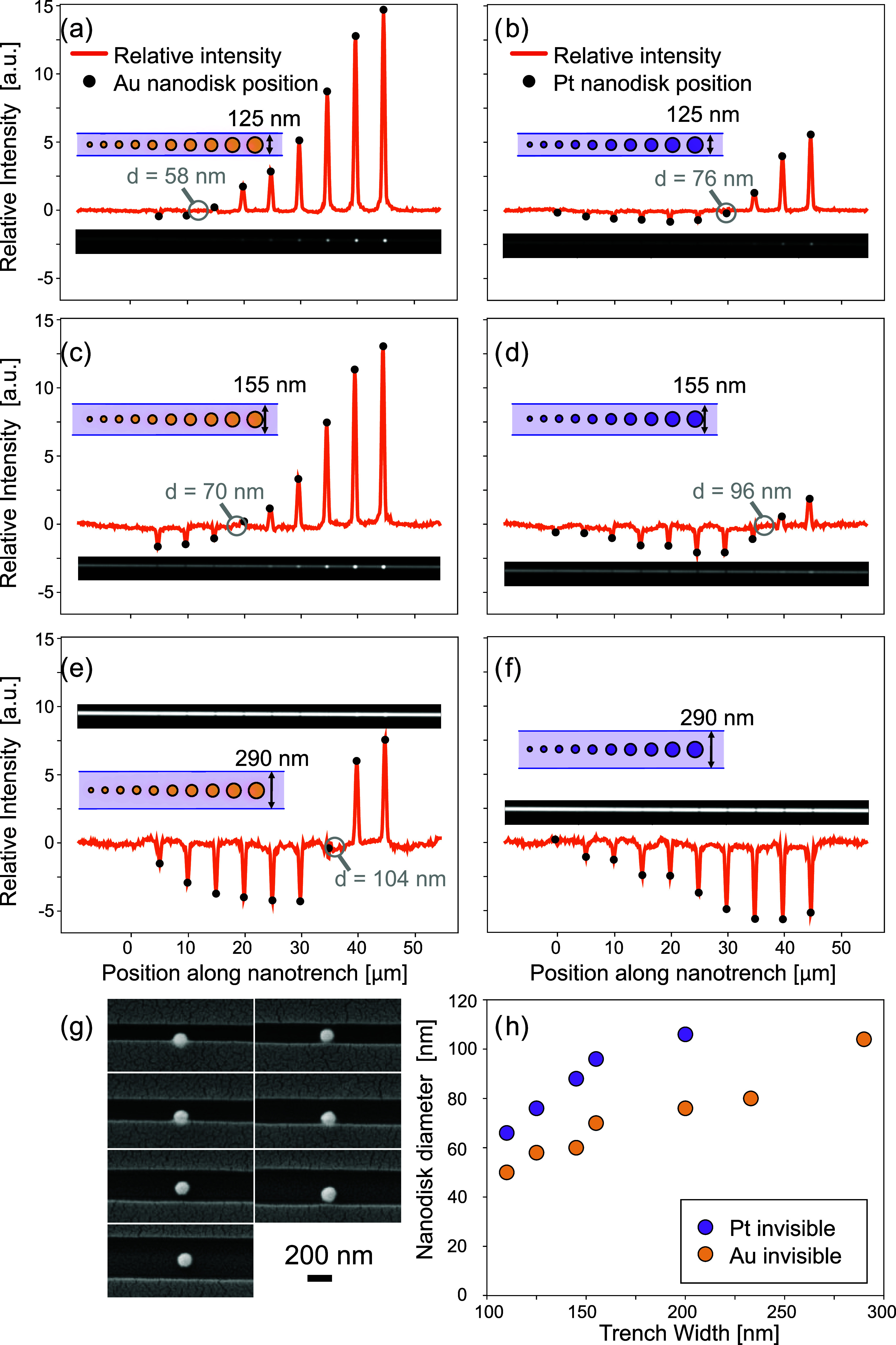
(a) Light scattering intensity of a Au nanodisk array located in a 125 nm wide nanotrench, measured at 600 ± 10 nm wavelength and plotted relative to the scattering intensity of the nanotrench at a position without a particle. The nanodisk diameter along the array varies from ∼30 nm to ∼120 from left to right in nine steps. The internanodisk distance is 5 μm. The gray circle marks an inferred spot along the particle array, between a “dark” particle to the left (56 nm), and the “bright” on the right (62 nm), at which the scattering contrast inversion occurs and at which a particle therefore would be completely invisible. The inset depicts an absolute dark-field scattering intensity image of the nanotrench with the Au nanodisk array inside. (b) same as (a) but for an array of Pt nanodisks. (c) and (d) same as (a) and (b) but for a 155 nm wide nanotrench. (e) and (f) same as (a) and (b) but for a 290 nm wide nanotrench. (g) Representative SEM images of nanotrenches with 7 different widths all containing a metal nanodisk. (h) Inferred nanodisk diameter at which scattering contrast inversion occurs for the Pt and Au systems in nanotrenches of different widths (see SI Figure S7 for raw data).

Having experimentally observed the distinct dependence of the scattering response of a nanoparticle inside a nanotrench on the width of said trench, it is interesting to again apply our theoretical formalism for a qualitative analysis and comparison. For this purpose, we calculated the relative scattering cross-section, σ_s,rel_, of Au and Pt spheroidal particles with a fixed 60 nm height and varying diameter, localized inside cylinders with 125 nm, 155 and 290 nm diameter, respectively (Figure 5a–c). In line with the experimental results of the nanodisk-nanotrench system (cf. Figure 4), an increase in cylinder diameter in the model shifts the scattering contrast inversion point, i.e., the point where σ_s,rel_ = 0, toward larger spheroids. In addition, when the cylinder width is changed, we also note a shift in diameter of the spheroid that produces the largest dark scattering contrast, i.e., exhibits the most negative σ_s,rel_ value – also here in good qualitative agreement with the experiment (cf. Figure 4). These trends are summarized in Figure 5d and corroborate the experimentally found strictly positive trend for both Au and Pt where the diameter of the “invisible” Au particle always is smaller than the corresponding Pt one when located in a nanotrench/cylinder of the same width. The wavelength in the calculations presented in Figure 5 is 600 nm (see corresponding calculations at 500 and 700 nm in SI Figure S7). As the final point, we also note that minor position variations of the nanodisks with respect to the nanochannel central axis are present in the experiments (cf. Figures 1c,d and 4g), as a consequence of the alignment accuracy of the EBL nanofabrication process. Given the good agreement between the analytical model and the experimental data in terms of overall trends, we argue that these variations only have a minor impact on overall scattering response of the system, in good agreement with FDTD simulations for dielectric nanoparticles inside a nanofluidic channel.^17^

**Figure 5:**
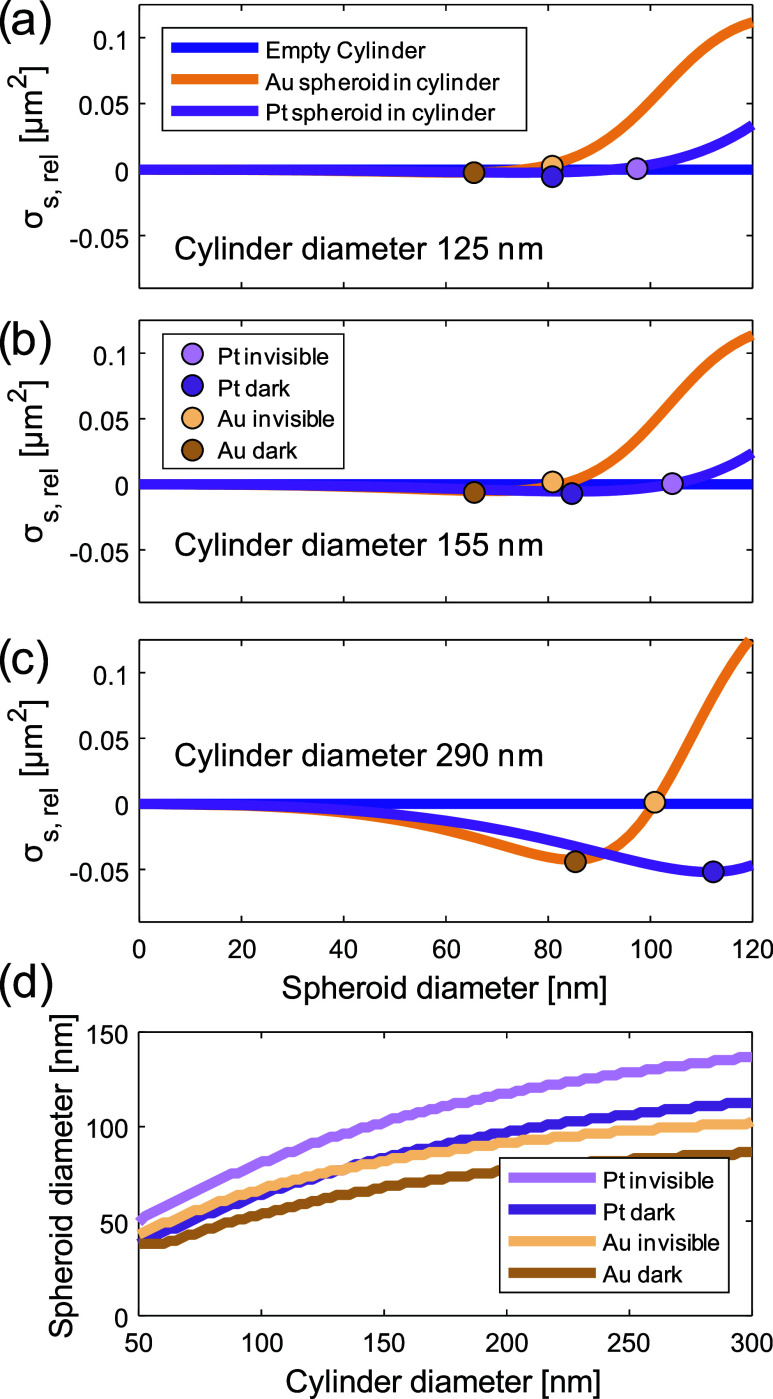
(a) Scattering cross sections of Au and Pt spheroids with different diameters inside a cylinder with 125 nm diameter, σ_s,rel_, calculated using our model for 600 nm irradiated light and plotted relative to the scattering cross section of the empty air-filled cylinder. The circular markers represent the spheroid particle diameters of invisible and darkest Au and Pt particle, respectively. (b) same as (a) but for a 155 nm wide cylinder. (c) same as (a) but for a 290 nm wide cylinder. (d) Calculated Au and Pt spheroid diameter at which the particle becomes invisible (σ_s,rel_ = 0), plotted as a function of cylinder diameter and together with the spheroid diameter at which the particle appears the darkest, i.e., exhibits the largest negative σ_s,rel_.

As the last step of this work and to demonstrate a first proof-of principle application of the discovered scattering contrast inversion phenomenon, we investigated how the light scattering signature of a single metal nanodisk inside a nanofluidic channel fully enclosed inside a SiO_2_ matrix can be dynamically tuned by exchanging the medium inside the nanochannel and thereby altering its RI. As evident from the analytical model developed above, the RI of the medium inside the nanochannel, *n*_c,_ will affect the scattering cross section of the nanochannel-nanodisk system (see SI Figure S4, S12). For this purpose, we micro and nanofabricated (see Methods for details) a fully functional fluidic chip comprised of an array of 150 nm wide and 70 nm deep nanochannels, which on both sides are connected to microfluidic channels that are 50 mm wide and 1.5 mm deep, and that can be accessed via inlet openings when mounting the fluidic chip on a custom sample holder (Figure 6a,b). Furthermore, we EBL-fabricated a linear array of Pt and Au nanodisks with different diameters and constant nominal 40 nm thickness and 5 mm inter-nanodisk distance into the nanofluidic channels, prior to hermetically sealing the chip by fusion-bonding an optically transparent Borofloat 33 glass lid (see Methods for details on fabrication). The chip was then mounted on a custom holder to enable the flushing of liquids through the fluidic system by means of applying pressure to the inlets using a Fluigent MFCS-EX pressure controller. During the liquid exchange experiment, the chip and holder were mounted on a dark-field microscope and illuminated by an LED light source through a ring illumination objective with a linear aperture placed in a 90° angle to the nanochannel. The aperture was used to suppress the light scattered from the nearby microchannel^14^ (see Methods for details). Two liquid exchange experiments were conducted to investigate the impact of the RI of the medium inside the nanochannel on the scattering contrast of the metal nanodisks (Figure 6c).

**Figure 6:**
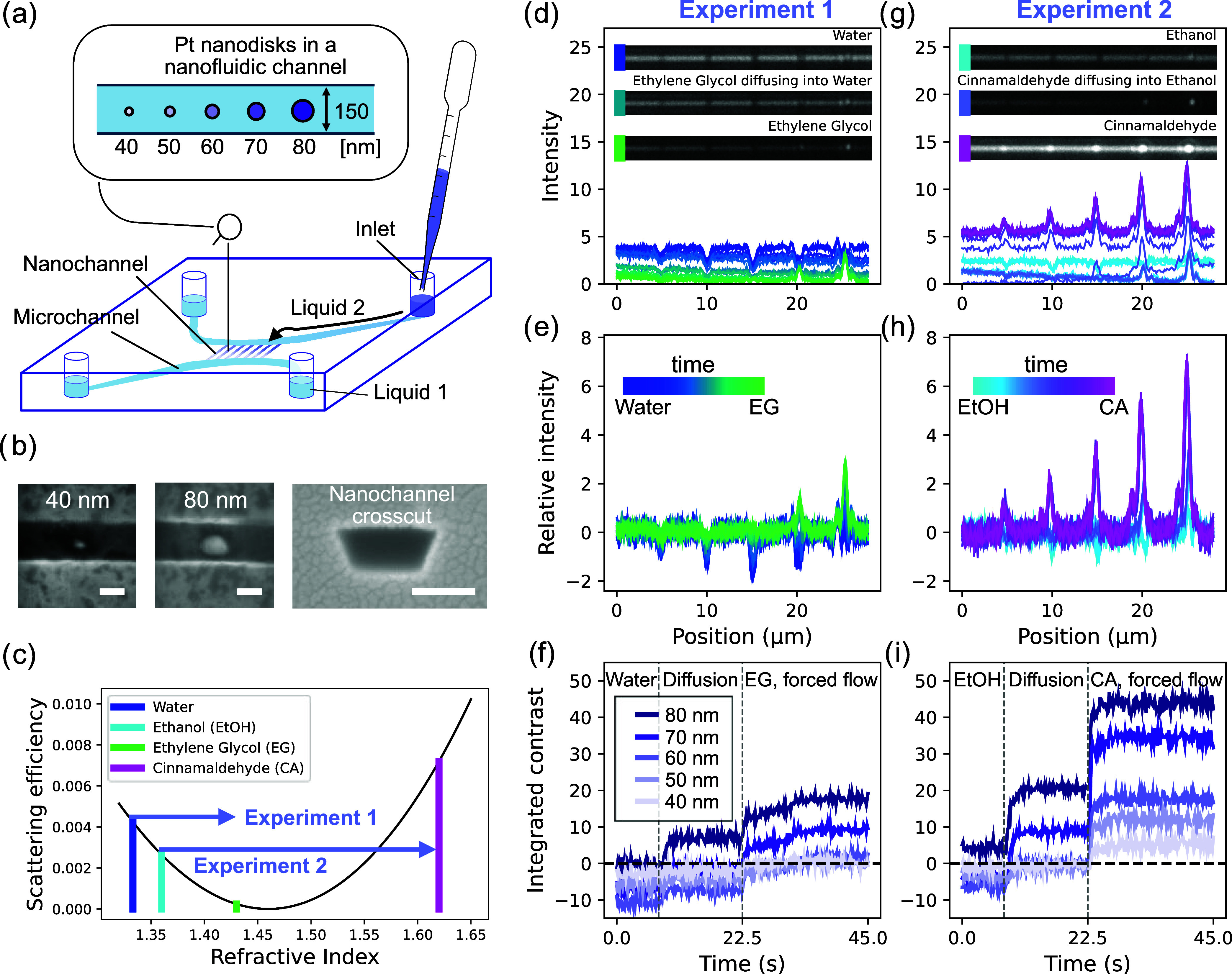
(a) Schematic depiction of the fully functional nanofluidic chip used for two experiments with liquids of different RIs. The inset schematically depicts five Pt nanodisks with diameters ranging from 40 to 80 nm that we EBL-nanofabricated within a 150 nm wide and 70 nm deep nanofluidic channel prior to hermetically sealing the chip by bonding of a Borofloat glass lid. (b) SEM images of a nanofluidic chip. The first two images display a top view of single Pt nanodisks prior to the bonding of the lid, and the third image shows a crosscut of a generic nanofluidic channel after the chip has been sealed. Note the complete and hermetic enclosure of the nanofluidic channel. The scale bars are 100 nm. (c) Scattering efficiency calculated for a homogeneous infinite cylinder with 150 nm in diameter embedded in SiO_2_ (*n*_SiO2_ = 1.46) as a function of the RI of the liquid medium inside the cylinder. Also indicated are the RI-transitions executed in experiments 1 and 2, respectively. (d) In experiment 1 the liquid content of the nanofluidic channel is exchanged from water to ethylene glycol and imaged with a dark-field microscope over the course of 45 s to extract the corresponding scattering intensity along each frame. The insets display dark-field scattering images of the nanochannel with the five nanodisks as the liquid content is exchanged. (e) The scattering intensities of the five Pt nanodisks inside the nanochannel in experiment 1 plotted relative to the scattering intensity of the nanochannel alone. Note the distinct inversion of scattering contrast from dark to bright for the two largest disks, while the smaller ones become invisible. (f) Time-resolved integrated scattering contrast of the five Pt nanodisks obtained during the liquid exchange from water to ethylene glycol in experiment 1. (g) In experiment 2 the liquid content of the nanofluidic channel is exchanged from ethanol to cinnamaldehyde and imaged with a dark-field microscope over the course of 45 s to extract the corresponding scattering intensity along each frame. The insets display dark-field scattering images of the nanochannel with the five nanodisks as the liquid content is exchanged. Note that in this experiment the RI of the liquid is altered from <*n*_SiO2_ to >*n*_SiO2_, and the system thus passes a stage where the scattering efficiency of the nanochannel is zero. (h) The scattering intensities of the five Pt nanodisks inside the nanochannel in experiment 2 plotted relative to the scattering intensity of the nanochannel alone. Note that the brightness of all particles increases significantly. (i)­Time-resolved integrated scattering contrast of the five Pt nanodisks obtained during the liquid exchange from ethanol to cinnamaldehyde in experiment 2.

In experiment 1, we exchanged the liquid in the nanochannels from water (*n*_water_ = 1.33) to ethylene glycol (*n*_EG_ = 1.43) over the course of 45 s. Specifically, we first established a water flow through the nanochannels by applying an overpressure to the chip inlet containing water. Next, the pressure was equalized on all inlets, allowing ethylene glycol to diffuse into the channels. Lastly, we established an ethylene glycol flow by applying pressure to the opposite side of the chip. Applying again our analytical model shows that by exchanging the liquid in this way, the RI inside the system is increased such that *n*_c_ approaches the RI of the embedding medium (*n*_SiO2_ = 1.46), which in turn reduces the scattering efficiency of the cylinder in the calculation (Figure 6c), and correspondingly of the nanochannel in the experiment (Figure 6d and Supporting Video 1 for the 5 Pt nanodisks and SI Section 4 for corresponding results obtained from the Au nanodisks). If we subsequently subtract the intensity along the collected dark-field images of the Pt nanodisks positioned in a nanochannel from the intensity of an empty channel, the relative intensity reveals an inversion of contrast, from dark to bright, for the two largest disks (70 and 80 nm), while the three smallest disks optically vanish (Figure 6e). This contrast change and inversion also become evident when tracking the integrated contrast i.e., the sum of the relative scattering intensity attributed to each nanodisk, as a function of time during the liquid exchange experiments (Figure 6f). Projecting the conditions of experiment 1 onto our calculations of a Pt spheroid placed in a cylinder, the change in relative scattering intensity can be attributed to the liquid RI approaching the RI of the SiO_2_ matrix. As ethylene glycol reduces the scattering efficiency of the cylinder, the interference contribution to the total intensity is reduced, and the relative scattering of the two-object system thus more closely resembles the scattering spectrum of the particle alone (see SI Figure S12).

To further explore how the interplay of scattering intensity of the nanochannel, nanodisk, and their interference can be tuned to enhance the contrast of a nanoparticle inside a nanochannel, we conducted a second liquid exchange experiment (Figure 6g–i and in Supporting Video 2). In this experiment, we investigated the contrast produced by a liquid with RI higher than the SiO_2_ matrix that the nanochannel is embedded in. Specifically, we let cinnamaldehyde (n_CA_ = 1.62) diffuse into ethanol (*n*_EtOH_ = 1.38) and again observed the effect of an inversion in the sign of the contrast as the RI of liquid in the nanochannel is increased. However, while the ethylene glycol filled channel in the first experiment induced a positive contrast for only the two largest Pt nanodisks while rendering the rest invisible, the cinnamaldehyde filled channel results in a strong positive contrast for all five studied nanodisks. Interestingly, this large contrast observed for cinnamaldehyde is not a consequence of a reduced interference term, as it was in the case of ethylene glycol, but can rather be explained by a change of sign for the interference contribution. Specifically, by introducing a liquid with *n* > *n*_SiO2_, the polarizability of the nanochannel assumes positive values (see SI Figure S12d for the corresponding calculated case of a cylinder), which in turn will result in constructive interference for all parts of the spectrum where the real part of the particle/nanodisk polarizability is positive, as can be deduced from eq 10.

Turning our attention back to the integrated contrast measured for the 5 Pt nanodisks presented in Figure 6f,i, it is worth noting that the largest scattering contrast of each disk depends on the liquid state in each experiment; in experiment 1 the largest contrast of the largest disk (80 nm) was achieved in the channel filled with ethylene glycol, while the three smallest disks (40–60 nm) exhibited their largest contrast in the water-filled channel. Meanwhile, in experiment 2 all disks attained their maximum contrast when immersed in cinnamaldehyde.

## Conclusions

In this work, we have demonstrated that the scattering of light by plasmonic metal nanodisks located in a nanotrench embedded in PMMA, or in the last part of this work in a fully enclosed nanofluidic channel embedded in a SiO_2_ matrix, is affected by the dimensions and optical properties of the nanodisk and the trench/channel, and importantly also by the interference of light scattered from the two. Studying first nanoparticles in nanotrenches that emulated fully enclosed nanofluidic channels, we found that by varying system parameters, such as the disk diameter and type of metal, the nanotrench width and the irradiated light wavelength, the optical contrast of the disks relative to an empty trench can be systematically tuned between bright, dark, and effectively invisible. Interestingly, all investigated systems displayed a reoccurring relation between optical contrast and disk diameter, where with increasing disk diameter the negative contrast, i.e., darker appearance than the trench, first increased, reached a minimum and then decreased to reach a point of invisibility, before a monotonously increasing positive contrast (disk brighter than trench) was observed. This trend was qualitatively also accurately captured with the derived analytical theoretical framework, where we modeled the scattering response of a nanodisk-nanotrench system with the scattering cross section from a diffraction limited spot containing a spheroidal particle localized inside a cylinder embedded in a homogeneous matrix. From our model, we found that the light scattering contrast of plasmonic particles inside nanofluidic systems depends on the real and imaginary parts of their polarizability, which are determined by particle parameters like geometry and material properties. Looking forward, more quantitative theoretical descriptions using for example electrodynamic simulations using, e.g., FDTD methods, can be attempted to shed further light on more intricate details of this effect, such as nanoparticle position inside the nanochannel, or nanoparticle and nanochannel geometry.

In the last part of our work, we applied the insights obtained from the nanotrench-nanodisk model system and the analytical model calculations to a fully functional nanofluidic system comprised of enclosed 150 nm wide and 70 nm deep nanochannels decorated with Pt and Au nanodisks of different size. We measured the relative scattering intensities of the particles when the nanochannel was filled with liquids with RIs varying from being smaller than the RI of the SiO_2_ matrix embedding the nanochannel to being larger the RI the SiO_2_ matrix. These experiments revealed that the light scattering contrast of single metal nanoparticles inside a nanofluidic channel can be tuned from bright to dark to invisible by varying the RI of the liquid inside the nanofluidic channel. Interestingly, in the RI < *n*_SiO2_ regime, the contrast is dictated by a RI-mediated reduction of the interference-term contribution to the scattering efficiency, whereas in the RI > *n*_SiO2_ regime, the contrast is dictated by a sign-change for the interference contribution to the total scattering.

Looking forward, we predict our findings to pave way for single particle nanofluidic experiments of processes, such as colloidal crystal growth, ligand – receptor binding events^33^ or surface reconstruction during catalysis,^34^ that by altering the scattering response of single particles inside nanofluidic channels, and therefore their optical contrast, can be studied under highly controlled conditions. We also anticipate that the contrast inversion phenomenon can be used to study fluids and their compositions in nanofluidic systems since such fluidic systems today are widely used in combination with aqueous solutions, organic solvents, acids, and electrolytes,^3,35,36^ where knowing their exact *local* composition is important.

## Supplementary Material







## Notes

Pt nanodisks: water - ethylene glycol exchange (Video S1) (MP4)
Pt nanodisks: ethanol - cinnamaldehyde exchange (Video S2) (MP4)
SEM imaging of nanotrenches and nanodisks, derivation of the total scattering cross section of a metal spheroid inside a cylinder, data analysis of nanotrench samples, and liquid exchange experiment (PDF)
