# Supplementary material for: Light Scattering Contrast Inversion of Single Metal Nanoparticles Inside a Nanofluidic Channel
Source: J Phys Chem C Nanomater Interfaces. 2026 Jul 28;130(31):10988–1000. doi: 10.1021/acs.jpcc.6c03991 (PMC13455110; doi:10.1021/acs.jpcc.6c03991)
Supplement: Supplementary file 1 [file jp6c03991_si_003.pdf]

Supplementary Information For

Light Scattering Contrast Inversion of Single Metal Nanoparticles inside a Nanofluidic Channel

Lova Wilske[1], Joachim Fritzsche[1], Barbora Špačková[2], Bohdan Yeroshenko[1], and Christoph Langhammer[1,]*

[1]Department of Physics and Astronomy, Chalmers University of Technology, SE-412 96 Göteborg, Sweden

[2] FZU - Institute of Physics of the Czech Academy of Sciences, 182 00 Prague 8, Czech Republic

Section S1. SEM imaging of nanotrenches and nanodisks

Scanning electron microscopy (SEM) was used to determine the nanodisk diameter and the width of the nanotrenches discussed in **Figure 1,** and **4**. Prior to the SEM analysis a 1 nm layer of gold was sputtered onto the sample to prevent charge build up from the electron beam. The SEM images of Au and Pt nanodisks in a 200 nm wide trench are presented in **Figure S1** together with the measured disk diameters. An investigation of nanotrench width is presented in **Figure S2**. An acceleration voltage of 7 kV was used, the magnification in each image is 50 kX and the working distance was 3.3 mm.

SEM images of nanodisks placed on an open SiO_2 surface are shown in **Figure S3**. The measurement was used to determine the nanodisk diameter given in **Figure 2b,c**. In the measurement, the acceleration voltage was set to 7 kV, magnification was 175 kX, and the working distance was 3.7 mm. No additional layer was sputtered prior to SEM.

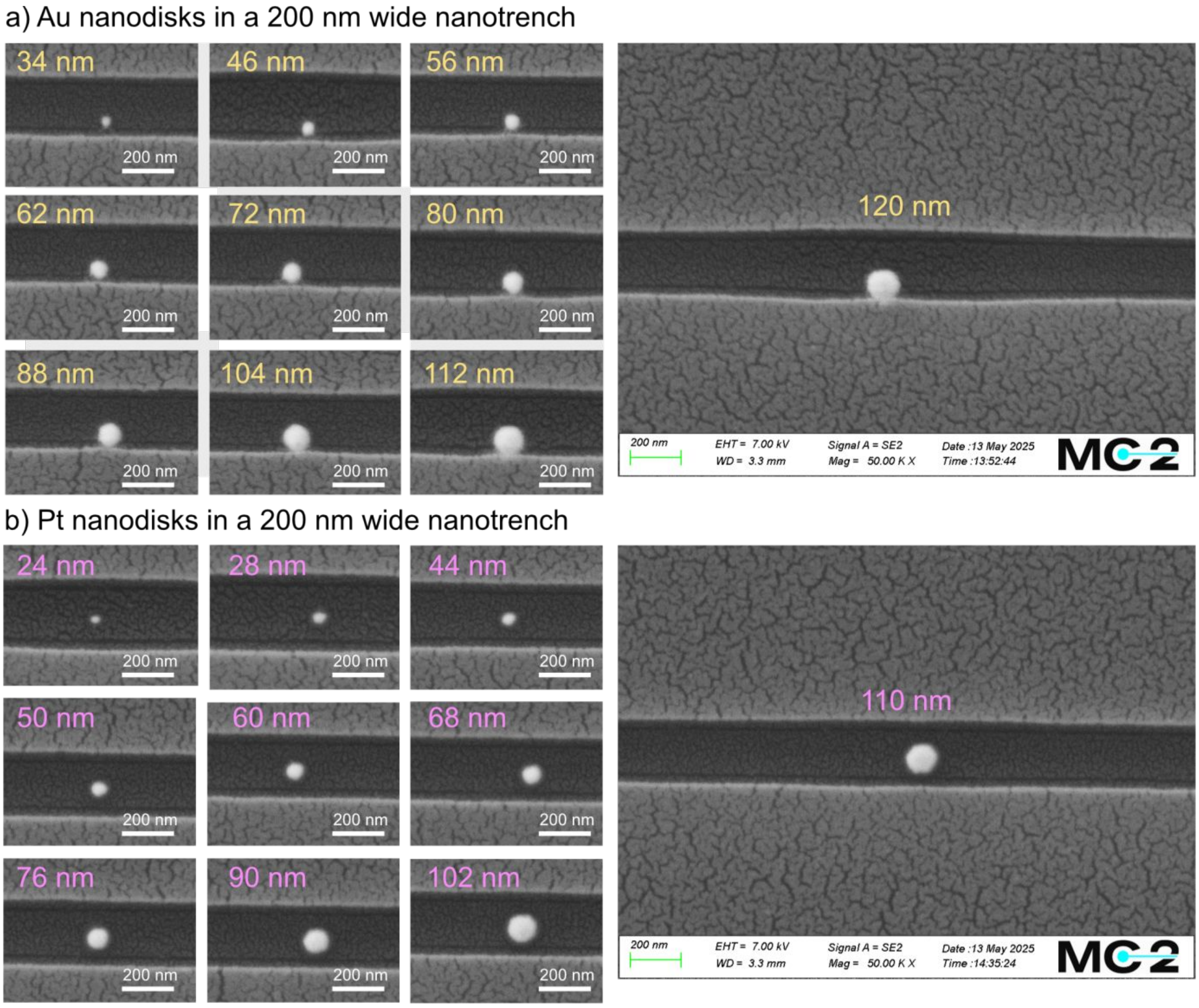

Figure S1. *SEM images of Au (a) and Pt (b) nanodisks in a 200 nm wide nanotrench. The diameter of each disk is indicated in each figure.*

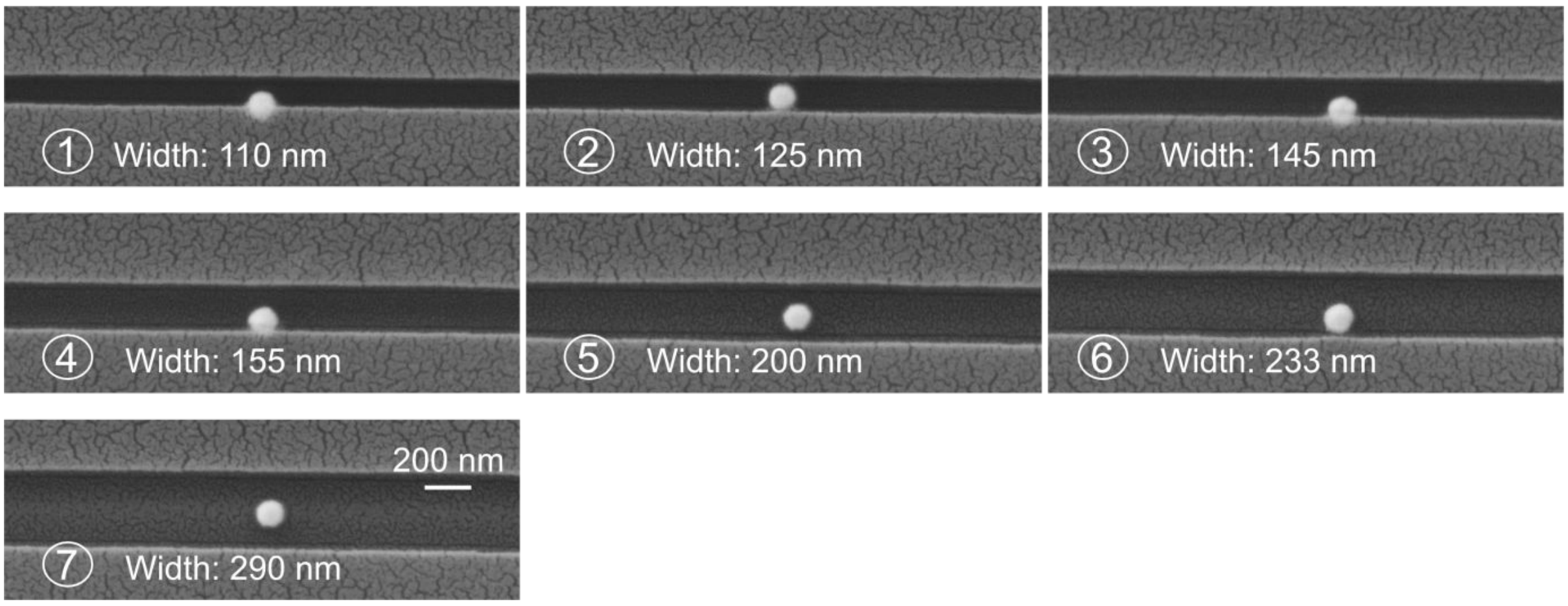

Figure S2. *SEM images of nanotrenches of various widths containing a single metal nanoparticle. The scalebar depicted in panel 7 is valid for all seven panels.*

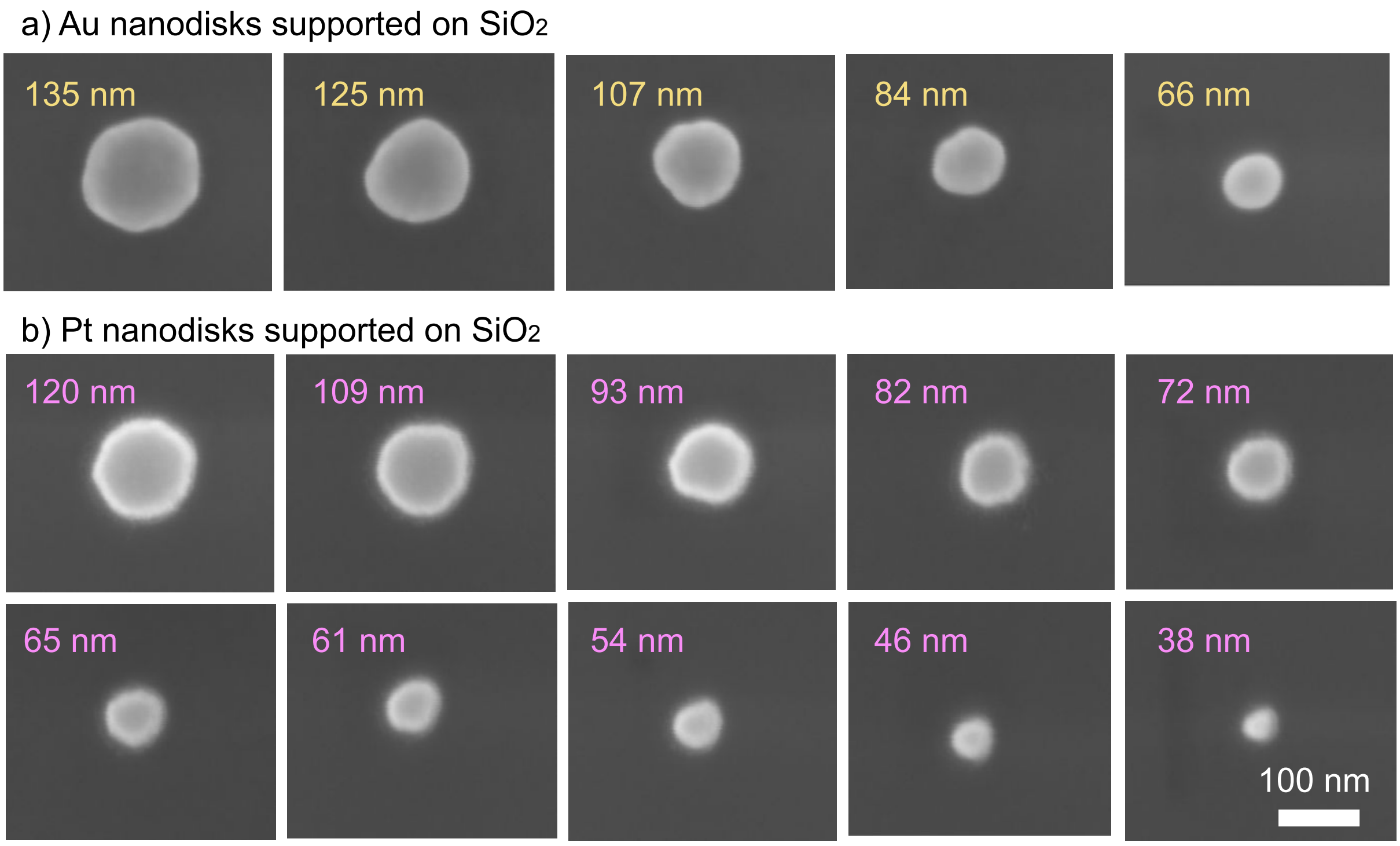

Figure S3. *SEM images of Au (a) and Pt (b) nanodisks. The diameter of each disk is indicated in each figure. The scalebar depicted in the last panel is valid for all panels.*

Section S2. Derivation of the total scattering cross section of a metal spheroid inside a cylinder

The total scattering cross section, $\sigma_{s,tot}$, of a diffraction limited spot containing a metal spheroidal particle located in a cylinder can be modelled as the scattering of an ideal dipole as

$$\sigma_{s,tot} = \frac{k^4}{6\pi}|\alpha_{tot}|^2 (S-1)$$

where $k = 2\pi n_o/\lambda$ is the wavenumber, and α_{tot} is the total polarizability of the studied volume, which we assume to be the sum of the polarizabilities of the cylinder, α_c, and the particle, α_p, as

$$\alpha_{tot} = \alpha_c + \alpha_p (S-2)$$

In the following section α_c and α_p are derived separately. We thereafter expand the expression of $\sigma_{s,tot}$ by inserting α_c and α_p and then finish the section by presenting additional results, as complement to **Figure 3**, using the model.

Polarizability of an infinitely long cylinder

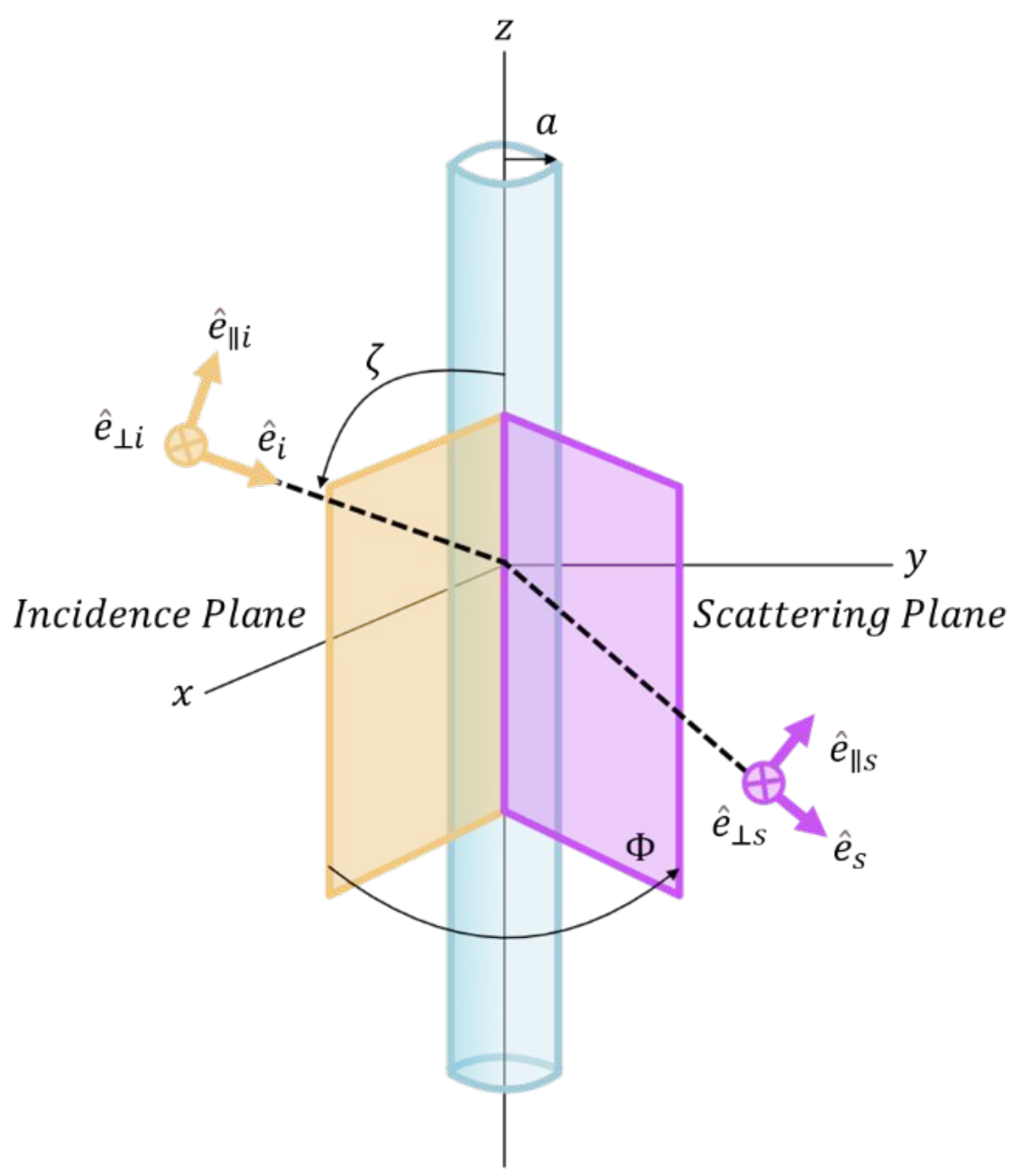

Figure S4. *Scattering of light by an infinitely long cylinder. The angle of illumination, denoted by ζ, is defined as the angle between the z and x axis. The illumination can be decomposed into light polarized parallel (TM mode) and orthogonal (TE mode) to the incident field as indicated in the figure.*

Let us first consider scattering from an infinitely long cylinder of radius a in a system of cylindrical coordinates, as depicted in **Figure S3**. Light scattering of plane waves impinging on a perfect homogenous infinitely long cylinder constitutes one of two systems for which there exists an exact analytical solution to the scattered electric and magnetic fields, $\mathbf{E}_s$ and $\mathbf{H}_s$. Incoming light can either be polarized parallel to the incident plane, orthogonal to the incident plane, or exhibit a mixed state of the two. The first case is referred to as transverse magnetic mode (TM), and the second as the transverse electric mode (TE). For TM polarization $\mathbf{E}_s$ and $\mathbf{H}_s$ are given by an infinite series of multipole radiation contributions as[1]

$$\mathbf{E}_s = -\sum_{n=-\infty}^{\infty} E_n\left(a_n M_n^{(H_n)} + b_n N_n^{(H_n)}\right)(S-3)$$

$$\mathbf{H}_s = \frac{ik}{\omega\mu}\sum_{n=-\infty}^{\infty} E_n\left(a_n M_n^{(H_n)} + b_n N_n^{(H_n)}\right)(S-4)$$

where $E_n = E_0(-i)^n/ksin\zeta$, ζ is the angle between the x and z axis, $k = 2\pi n_o/\lambda$ is the wavenumber, n_o the RI of the medium outside the cylinder, a_n and b_n are scattering coefficients, $M_n^{(H_n)}$ and $N_n^{(H_n)}$ are cylindrical vector harmonics based on the outgoing Hankel function, ω is the angular frequency, and μ the permeability of the surrounding medium. From $\mathbf{E}_s$ and $\mathbf{H}_s$, the scattering cross section of the cylinder, σ_s, can be expressed as the flow of energy, attributed to scattering, over an area Ω far from the cylinder as

$$\sigma_s = \frac{1}{I_i}\int_{\Omega}\frac{1}{2}Re\left(\mathbf{E}_s \times \mathbf{H}_s^*\right)d\Omega(S-5)$$

The scattering cross section of a fraction of a cylinder, $\sigma_{s,c}$, can be expressed in terms of the scattering efficiency of an infinitely long cylinder multiplied by the geometrical cross section of the cylinder fraction of interest

$$\sigma_{s,c} = 2alQ_s(S-6)$$

Here, l is the length along the cylinder over which the scattering cross section is studied. The scattering efficiency is a unitless parameter, that when scattered light from *every* angle around the cylinder is considered, can be expressed by the scattering coefficients of the following form

$$Q_{s,TM} = \frac{2}{x}\left(|b_{0,TM}|^2 + 2\sum_{n=1}^{\infty}|a_{n,TM}|^2 + |b_{n,TM}|^2\right)(S-7)$$

$$Q_{s,TE} = \frac{2}{x}\left(|a_{0,TE}|^2 + 2\sum_{n=1}^{\infty}|a_{n,TE}|^2 + |b_{n,TE}|^2\right)(S-8)$$

where $x = ka$ is a size parameter. To obtain a simplified expression of the scattering efficiency, the following approximations and boundary conditions will be applied: Within the Rayleigh regime, where the cylinder radius is much smaller than the incidence wavelength ($a \ll \lambda$), the radiation from the dipole mode ($n = 1$) will dominate the total scattering of the system, and we shall from hereon discard the higher order multipoles. As a second boundary condition on the system, the refractive index (RI) of the cylinder material and its surrounding medium will be restricted such that the relative RI, $m = n_c/n_o$, is close to 1. If we now only consider incidence at an angle normal to the cylinder length the scattering coefficients can be approximated as[1]

$$a_0 \approx \frac{-i\pi x^4(m^2-1)}{32},\ b_0 \approx \frac{-i\pi x^2(m^2-1)}{4},$$

$$a_1 \approx \frac{-i\pi x^2}{4}\frac{m^2-1}{m^2+1},\ b_1 \approx \frac{-i\pi x^4(m^2-1)}{32}(S-9)$$

and the scattering efficiency corresponding to TM and TE polarized light thus become

$$Q_{s,TM} \approx \frac{\pi^2 x^3}{8}(m^2-1)^2(S-10)$$

$$Q_{s,TE} \approx \frac{\pi^2 x^3}{4}\left(\frac{m^2-1}{m^2+1}\right)^2(S-11)$$

Considering only the scattering of light observed along a fraction of length l along the cylinder illuminated by unpolarized light, the scattering cross section becomes

$$\sigma_{s,c} = 2al\frac{1}{2}\left(Q_{s,TM}+Q_{s,TE}\right) = \frac{A^2k^3l}{4}(m^2-1)^2\left(\frac{1}{2}+\frac{1}{(m^2+1)^2}\right)(S-12)$$

where $A = \pi a^2$ is the area och the cylinder cross section. By rearranging the factors, $\sigma_{s,c}$ can be expressed such that the polarizability, α_c (in units of m^3), can be extracted as

$$\sigma_{s,c} = \frac{k^4}{6\pi}|\alpha_c|^2(S-13)$$

$$\alpha_c = \sqrt{\frac{3\pi}{2}\frac{A\sqrt{l}}{\sqrt{k}}}(m^2-1)\sqrt{\frac{1}{2}+\frac{1}{(m^2+1)^2}}(S-14)$$

If we set $l = k^{-1}$, and substitute $k = l^{-1}$, the spectrum for a cylinder of 200 nm diameter embedded in a SiO_2 matrix, filled with air and illuminated by unpolarized light applied perpendicular to the cylinder can be calculated as depicted in **Figure S4**. By subsequently repeating the calculations a cylinder filled with water and ethylene glycol, we find that the scattering cross section is significantly decreased as the difference between n_c, and n_o, is reduced (**Figure S4a**). **In Figure S4b**, we also show the respective contributions of TM and TE polarizations to the scattering cross section spectrum of a water filled cylinder. It is also worth commenting on the fact that a cylinder of 200 nm diameter is nearing the limit for which the approximation in eq S-9 is valid, and that the results in **Figure S4** therefore should be used qualitatively, rather than quantitatively.

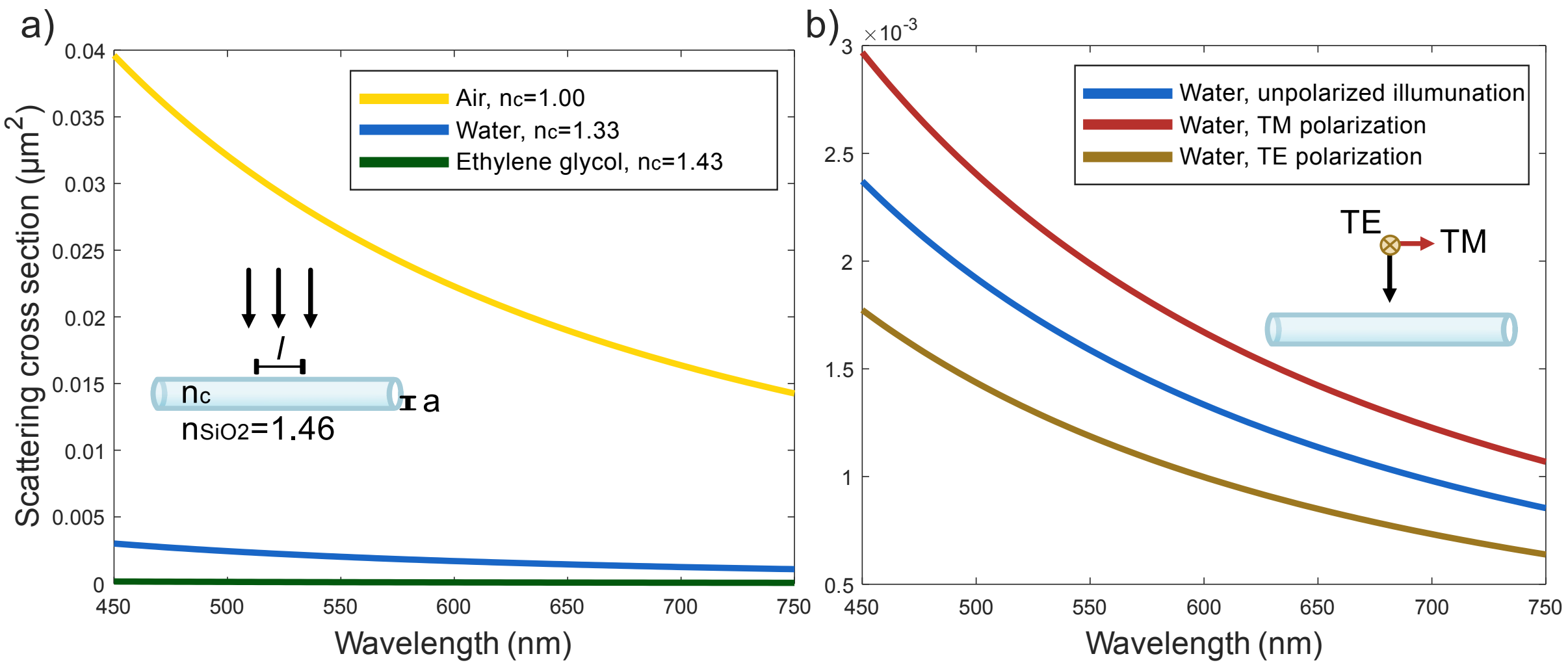

Figure S5*. a) Calculated scattering cross section spectrum for perpendicular illumination of unpolarized light onto an infinitely long homogenous cylinder of diameter 200 nm (a=100 nm), analyzed over a length $l = \lambda/2\pi n_o$, filled with air (n_c= 1.00), water (n_c= 1.33), and ethylene glycol (n_c= 1.43), respectively, and embedded in SiO*$_2$ *(n_o = 1.46)*[2]*. The inset illustrates perpendicular illumination of a cylinder, where the radius a, and length l are marked. The scattering cross section notably decreases as the gap between n_c and n_o is reduced. b) The TM and TE contributions to the scattering cross section of a water-filled cylinder of the same dimensions. The inset shows the direction of the respective polarization in relation to the cylinder.*

Polarizability of a spheroid

As the next step, we turn our attention to the scattering of light from metal ellipsoidal particles of radii a_1,a_2,a_3. For a homogeneous ellipsoid with $a_{1,2,3} \ll \lambda$, the scattering cross section can be modeled as an ideal dipole

$$\sigma_{s,p} = \frac{k^4}{6\pi}|\alpha_p|^2 \quad (S-15)$$

where α_p is the polarizability of the ellipsoid that within the modified long wavelength approximation (MLWA)[3,4] can be expressed as

$$\alpha_p = \frac{\alpha}{1 - i\frac{k^3}{6\pi}\alpha - \frac{k^2}{2\pi d}\alpha} \quad (S-16)$$

and where

$$\alpha = V_p \frac{n_p^2 - n^2}{n^2 + L_j\left(n_p^2 - n^2\right)} \quad (S-17)$$

with V_p being the particle volume, n_p its complex refractive index, n the effective refractive index of the environment (widely approximated as $n = (n_{SiO2} + n_{Air})/2$ for glass-supported nanoparticles), and L_j is a geometrical factor where j=1, 2, and 3, designates the axis of the coordinate system along which an electric field is applied parallel to. The axis along the largest radius is assigned j=1 (radius a_1) and the axis along the shortest radius is assigned j=3 (radius a_3). The geometrical factor along the longest axis for the case of prolates, oblates and spheres, respectively can be expressed as

$$L_{1,prolate} = \frac{1-e^2}{e^2}\left(-1+\frac{1}{2e}ln\frac{1+e}{1-e}\right)(S-18)$$

$$L_{1,oblate} = \frac{\sqrt{(1-e^2)/e^2}}{2e^2}\left(\frac{\pi}{2}-tan^{-1}\left(\sqrt{(1-e^2)/e^2}\right)\right)-\frac{(1-e^2)/e^2}{2}$$

$L_{sphere} = \frac{1}{3}$where $e = \sqrt{1-a_3^2/a_1^2}$ and designates the eccentricity of the spheroid.

In **Figure S5** modeled scattering cross section spectra, and the real and imaginary part of the polarizability are displayed. The particles have a constant height of 60 nm and diameters varied between 20-120 nm, to resemble the metal nanodisks fabricated within nanotrenches in the experiment. Incident light is applied along the vertical axis (the height of the particle) as illustrated in the inset of **Figure S5**. Here, it is worth noticing the large discrepancy between measured dark-field spectra (**Figure 2b**) of the lithography fabricated Pt nanodisks and the MLWA modeled spectrum of perfect Pt spheroids (**Figure S5b**), that can be explained by the geometry differences between the systems, both in terms of particle shape and illumination angle.

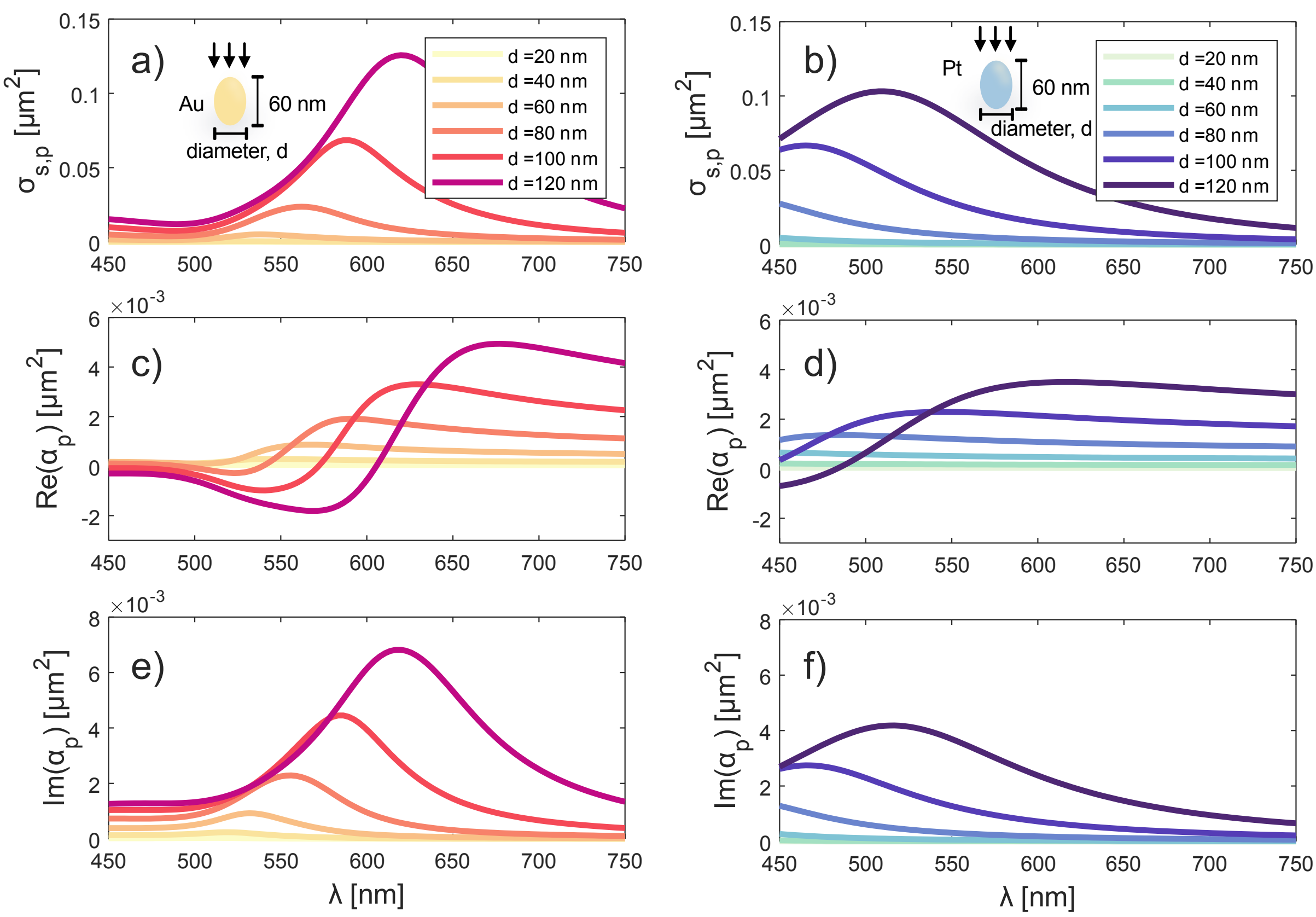

Figure S6. *Modeled scattering cross section spectra of Au (a) and Pt (b) metal spheroids. The modeled spheroids are 60 nm height and exhibit diameters varying from 20-120 nm. They are as shown in the inset. The RIs of the metals are adopted from Johnsson and Christy[5] (Au), and Werner[6] (Pt). The RI of the surrounding medium is defined as* $n = (n_{SiO2} + n_{Air})/2$ *to emulate the spheroid being localized on an* SiO_2 *support, as successfully done in earlier works on similar particles[7,8]. The real (c-d) and the imaginary (e-f) part of the polarizability of the same set of Au and Pd spheroids.*

Total scattering cross section

With α_c and α_p at our hands we can now expand eq. S-1

$$\sigma_{s,tot} = \frac{k^4}{6\pi}|\alpha_c + \alpha_p|^2 = \frac{k^4}{6\pi}\left(|\alpha_c|^2 + |\alpha_p|^2 + 2Re\left(\alpha_c\,\alpha_p^*\right)\right)(S-19)$$

If $\alpha_c = Re(\alpha_c)$ is assumed (which means that the refractive index of the cylinder only has a real part), using eq. S-13, S-15, and S-19 we obtain

$$\sigma_{s,tot} = \sigma_{s,c} + \sigma_{s,p} + 2sign\left(\alpha_c\alpha'_p\right)\sqrt{\sigma_{s,c}\sigma'_{s,p}}(S-20)$$

where $\alpha'_p = Re\left(\alpha_p\right)$ and $\sigma'_{s,p} = \frac{k^4}{6\pi}|\alpha'_p|^2$. The assumption of $\alpha_c = Re(\alpha_c)$ holds for most gases and liquids, such as water and organic solvents, in the visible spectral range. However, if for example dyes or other light-absorbing liquids/solutions with a complex refractive index[9] are to be used, the model needs to be adjusted. This adjustment is, however, beyond the scope of this work.

Additional results of the model

With the derived expressions of α_c and α_p at our hands, we can employ eq S-1 to calculate the total scattering cross section of a spheroid-cylinder system. In **Figure S6**, we present calculated relative scattering cross sections ($\sigma_{s,rel} = \sigma_{s,tot} - \sigma_{s,c}$) at 500 nm and 700 nm irradiated wavelength, respectively, for the same system as presented in **Figure 5** in the main text for 600 nm irradiated wavelength.

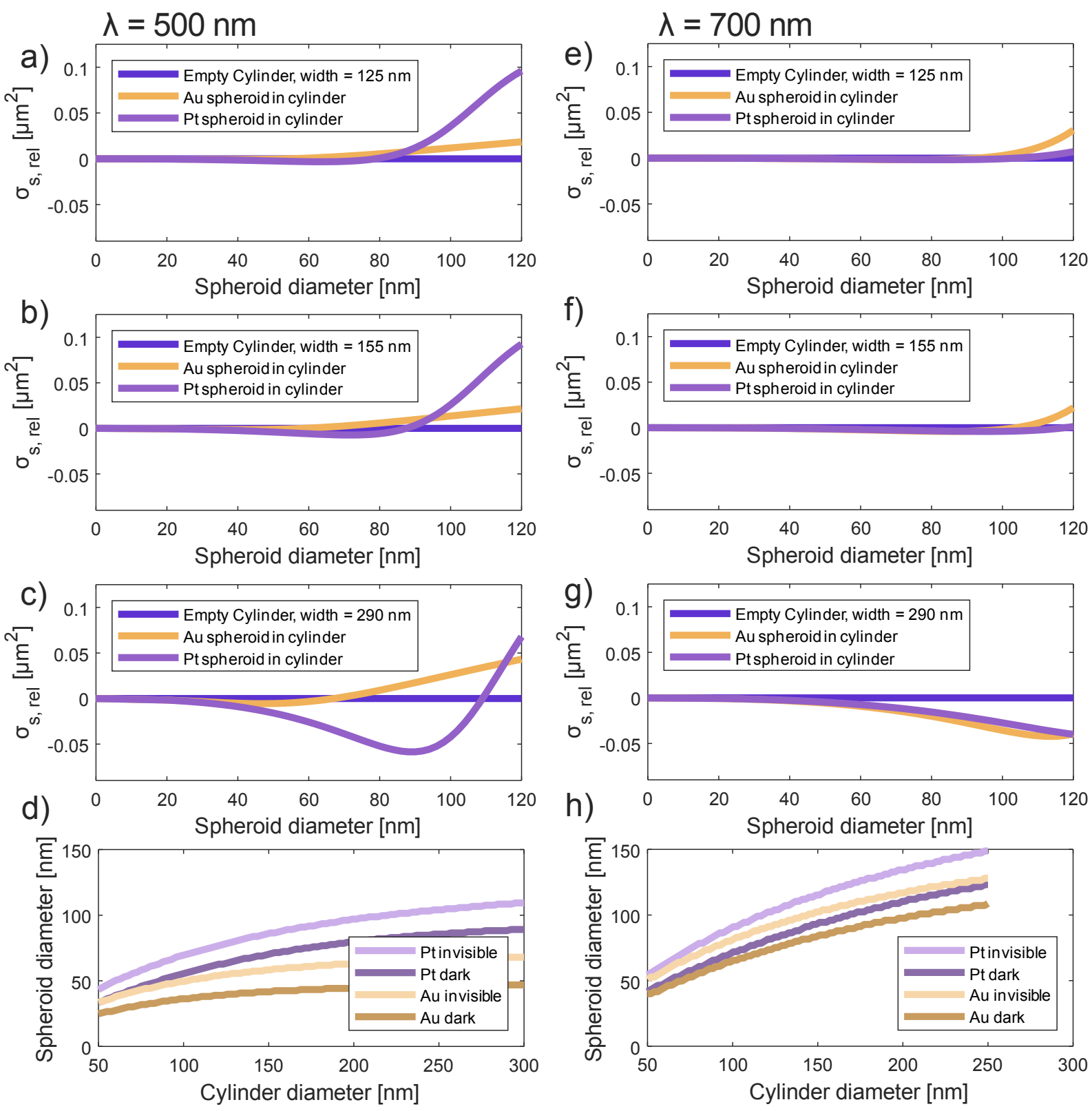

Figure S7. *a) Scattering cross sections of Au and Pt spheroids with different diameters inside a cylinder with 125 nm diameter (same as in **Figure 5** in main text) calculated using our model and plotted relative to the scattering cross section of the empty air-filled cylinder for 500 nm irradiated light, $\sigma_{s,rel}$. The circular markers represent the spheroid diameter of invisible and darkest Au and Pt particle, respectively. b) same as a) but for a 155 nm wide cylinder. c) same as a) but for a 290 nm wide cylinder. d) Calculated Au and Pt spheroid diameter at which the particle becomes invisible ($\sigma_{s,rel}$=0) as a function of cylinder diameter plotted together with the spheroid diameter at which the particle appears the darkest, i.e. exhibits the most negative $\sigma_{s,rel}$. The calculation was performed for a 500 nm wavelength. e-h) correspond to the same calculations as a-d) but for a 700 nm wavelength.*

Section S3. Data analysis of the nanotrench sample

The nanotrenches sample consists of nanotrenches with 8 different widths; 110, 125, 145, 155, 200, 233, 290 nm and one set of smaller trenches that were irregular in width and therefore excluded from the analysis. Each set of trenches was repeated 3 times on the sample with a constant distance of 5 μm between each trench. A schematic overview of the sample (**Figure S7a**) displays the design where one trench in each set is empty, the next is decorated with a size gradient array of 10 Au nanodisks and the third with 10 Pt nanodisks. The sample was imaged in a dark-field microscope in reflection mode using polychromatic white LED light. Bandpass filters transmitting light of 500 $\pm$ 10 nm, 600 $\pm$ 10 nm, and 700 $\pm$ 10 nm wavelength were, subsequently, placed in the collection pathway to acquire the images presented in **Figure S7b-d**. The 3 images were thereafter normalized by bright-field images of a Ag mirror, acquired using the same band pass filters. From **Figure S7b-d**, several regions of interests (ROIs) were selected to study the nanodisk arrays in each nanotrench separately. Each of these ROIs spanned 64.2×4.9 μm^2 with the nanotrench located in the middle. By summing up the intensity count of all pixels along the trench, an intensity profile of each ROI was obtained. Next, the *relative* intensity along the trench, i.e., the intensity contribution from the nanodisks and nanodisk-trench interference, was calculated by taking the following steps: from the nanodisk-free sections of the intensity attributed to the trench alone was retrieved. Since the nanotrench intensity varies slightly along the 64.2 μm analyzed section (likely due to inhomogeneous illumination and sample mounting), we approximated this intensity variation to be linear. The linear nanotrench intensity was then subtracted from the total intensity of the profile. The relative intensity trace of the Au and Pt nanodisk arrays from 500 $\pm$ 10 nm, 600 $\pm$ 10 nm, and 700 $\pm$ 10 nm scattered light is presented in **Figure S8, Figure S9**, and **Figure S10** respectively.

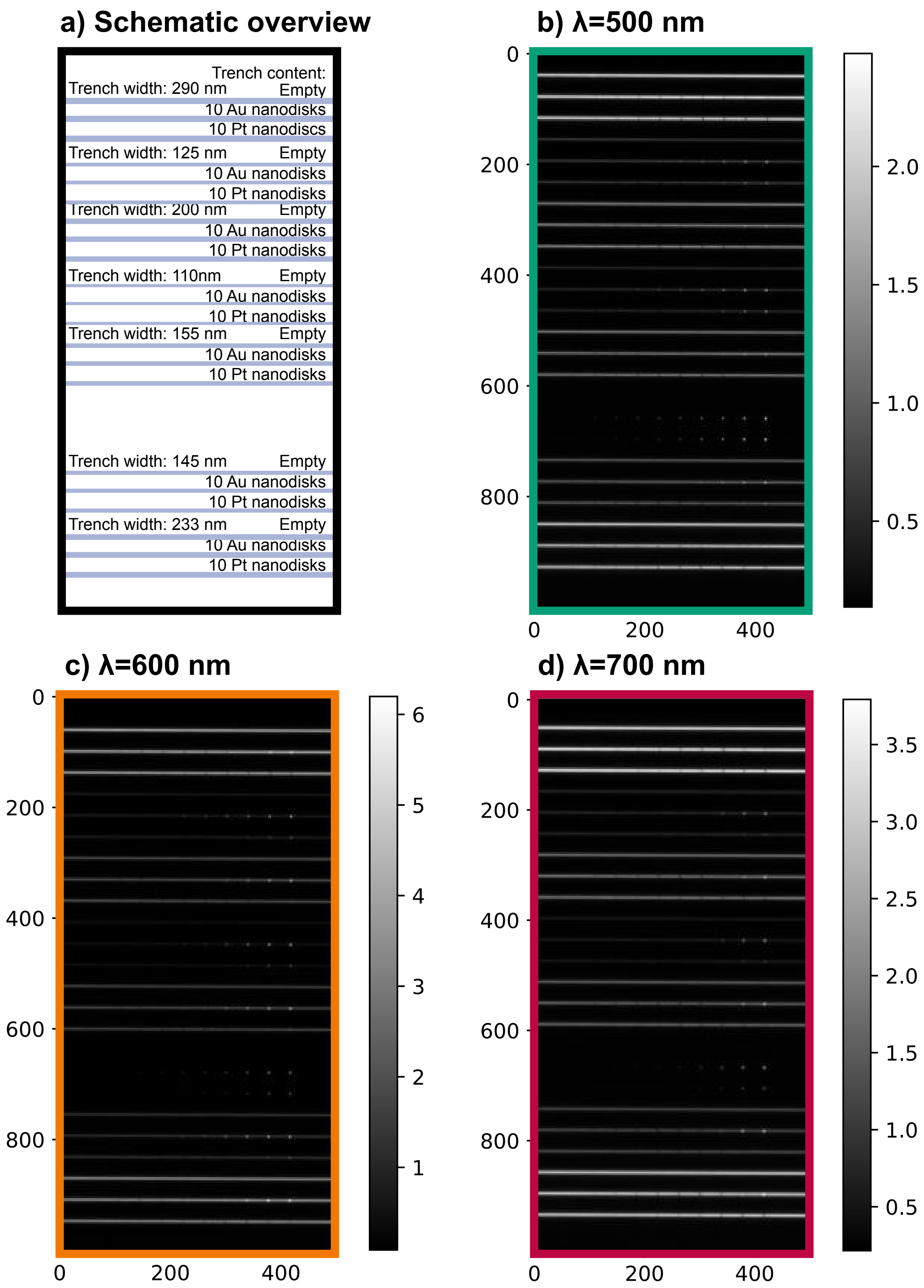

Figure S8. *a) Schematic illustration of the design of a chip constituting of trenches made of PMMA and metal nanodisk arranged in size gradient arrays of 10 particles from small to large. b) Dark-field image of the sample depicted in a), imaged for light of a 500* ± 10 *nm wavelength and normalized to a bright field reference image. The color bar shows the intensity of the scattering in the image. The image spans an area of 65 µm* $\times$ *133 µm. c) same as b) but imaged at 600* ± 10 *nm. d) same as b) but imaged at 700* ± 10 *nm.*

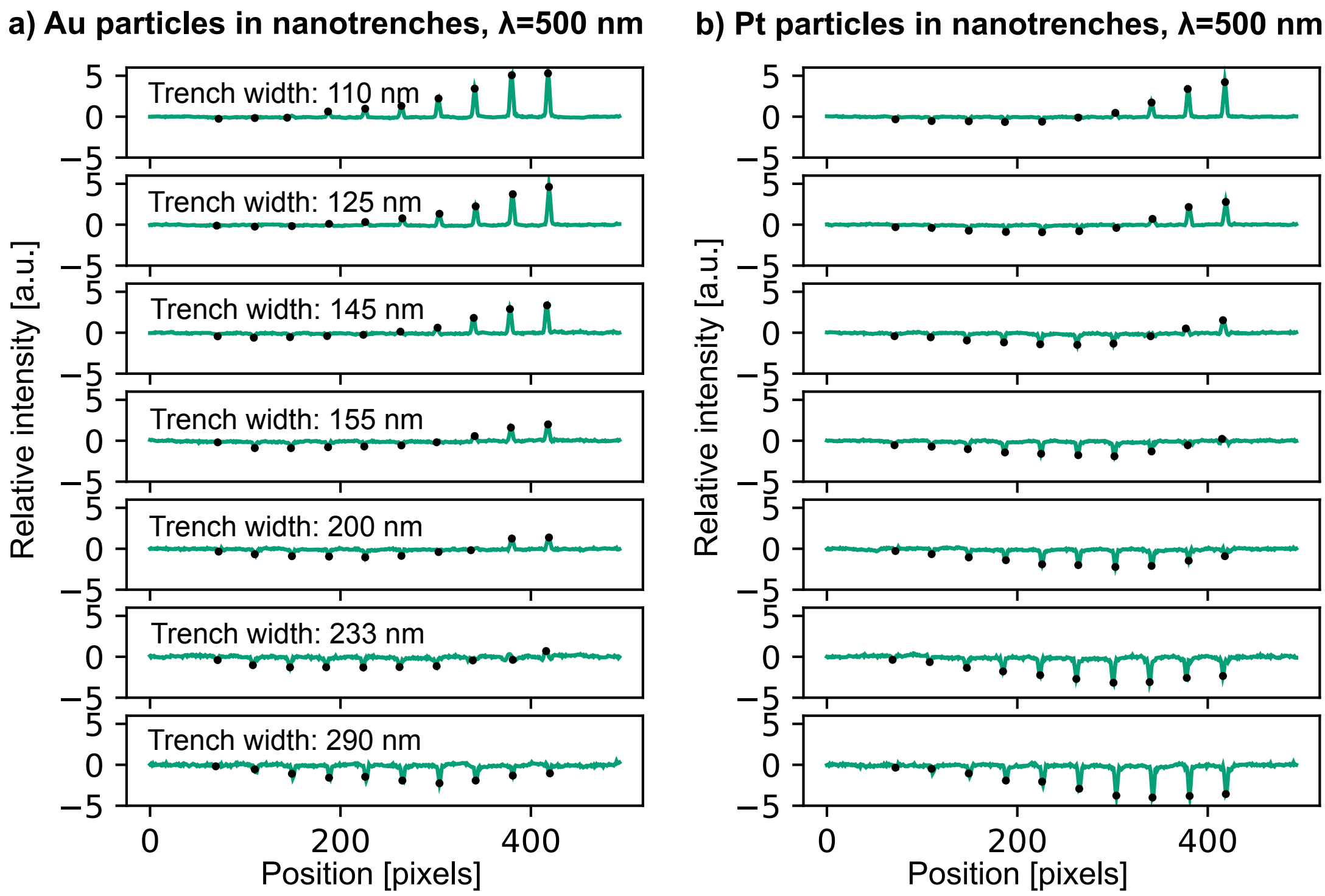

Figure S9. *a) Light scattering intensity of the Au nanodisk array measured for PMMA nanotrenches of different widths at 500 ± 10 nm irradiated wavelength plotted relative to the scattering intensity of the nanotrench at a position without particle. The black circles mark the position of a nanodisk. The trenches are studied along an image of 500 pixels which correspond to 65 µm. b) Same as a) but for the Pt nanodisk array.*

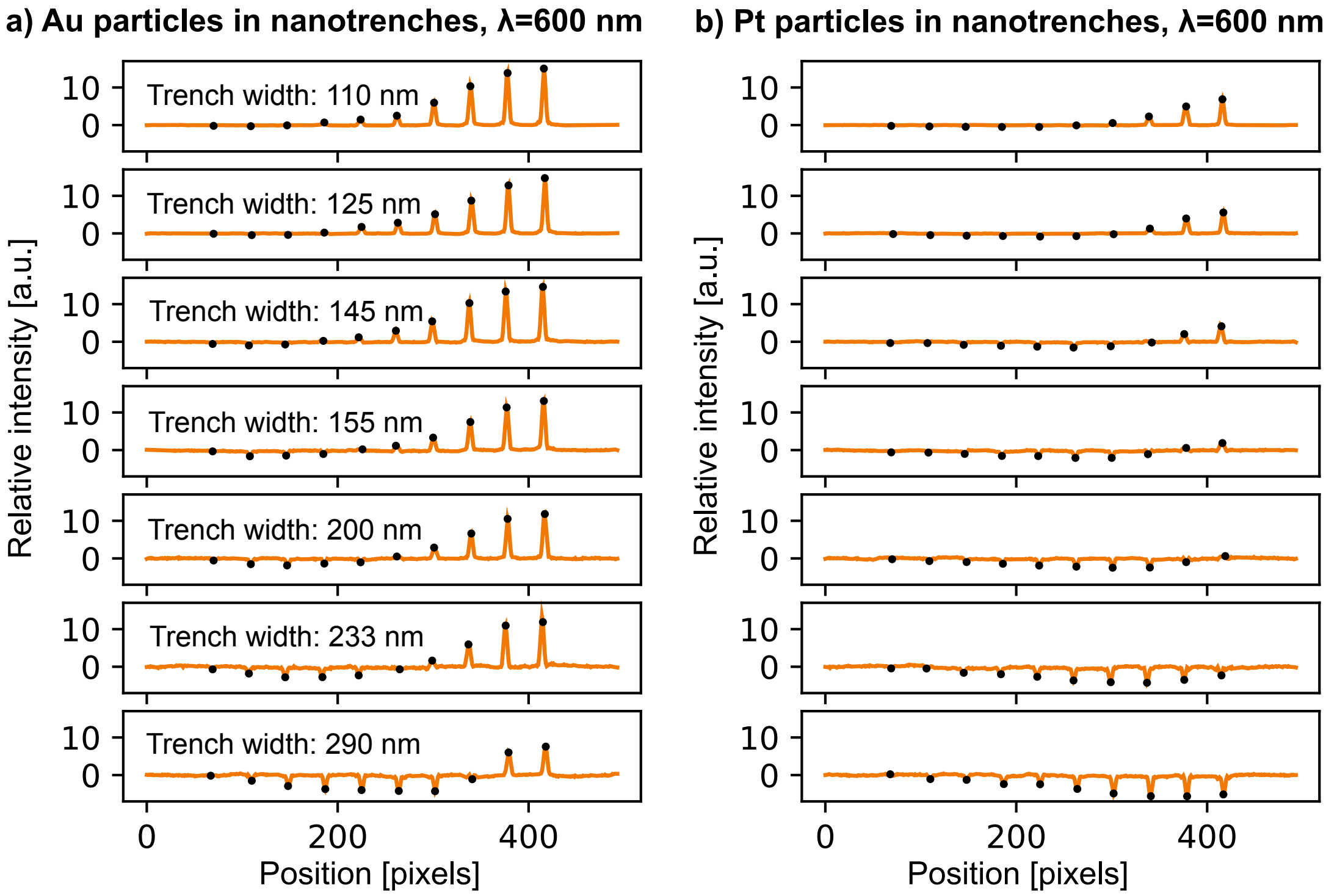

Figure S10. *a) Light scattering intensity of the Au nanodisk array measured for PMMA nanotrenches of different widths at 600 ± 10 nm irradiated wavelength plotted relative to the scattering intensity of the nanotrench at a position without particle. The black circles mark the position of a nanodisk. The trenches are studied along an image of 500 pixels which correspond to 64.9 µm. b) Same as a) but for the Pt nanodisk array.*

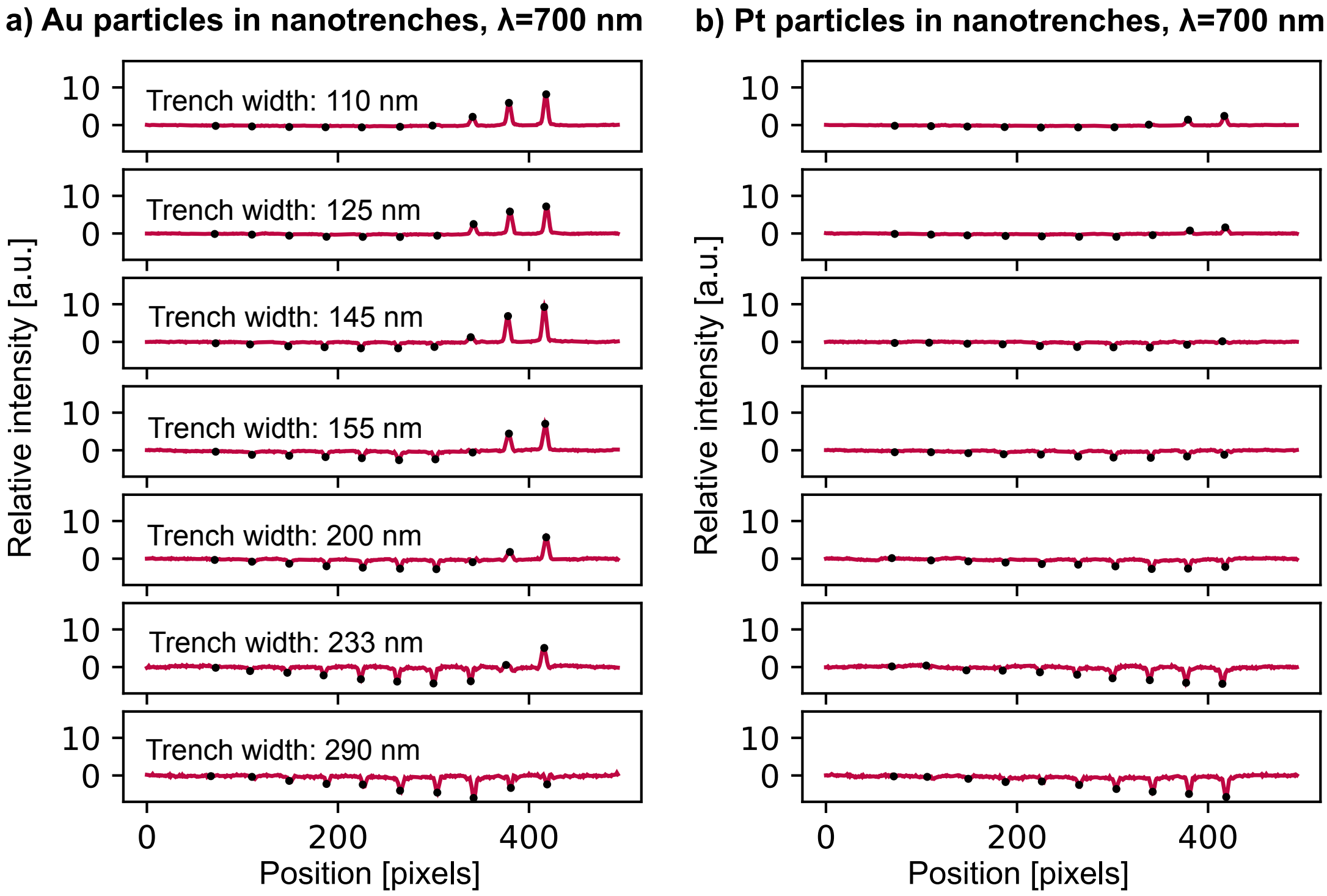

Figure S11. *a) Light scattering intensity of the Au nanodisk array measured for PMMA nanotrenches of different widths at 700 ± 10 nm irradiated wavelength plotted relative to the scattering intensity of the nanotrench at a position without particle. The black circles mark the position of a nanodisk. The trenches are studied along an image of 500 pixels which correspond to 64.9 µm. b) Same as a) but for the Pt nanodisk array.*

Section S4. Liquid exchange experiment

To design and interpret the data of the liquid exchange experiments presented in **Figure 6**, we modeled the scattering cross section of several metal spheroid-cylinder systems. Using the formalism derived for eq S-1, we found that the inner medium of the cylinder plays a crucial role in the particle contrast, i.e. the magnitude and sign of the particle scattering relative to the cylinder. In **Figure S12** we present the spectra for one interesting system comprised of an 80 by 40 nm Pt spheroidal particle embedded in a 150 nm cylinder that is filled with mediums with a range of RIs corresponding to water and to cinnamaldehyde. Here it can be noted that the sign of cylinder polarizability (**Figure S12d**) and the real part of the Pt particle polarizability (**Figure S12f**), that in turn govern the sign on the interference term (**Figure S12b**), varies greatly between the liquids.

The same two liquid exchange experiments conducted for Pt nanodisks in **Figure 6** were also conducted in a nanochannel decorated with 5 Au nanodisks (nominal diameters ranging from 40-80 nm). The spectra for a Au spheroidal particle placed in a cylinder is presented in **Figure S13**. An analysis of the dark-field scattering images collected during the experiments with Au nanodisks is presented in **SI Figure S14** and qualitatively shows a similar response of contrast inversion as was the case of Pt nanodisk. However, quantitatively the integrated contrast of the Au disks is generally smaller than for the Pt nanodisks. The difference in integrated contrast between the two systems can be understood by comparing the interference-term spectrum of Au and Pt spheroids placed cylinder, see **SI Figure S12** and **S13**. Here it can be noted that the calculated interference term for the Au particle-cylinder system (**SI Figure S13b**) changes in sign around the middle of the visible spectra which reduce the overall significance of the interference term when the system is illuminated by white light.

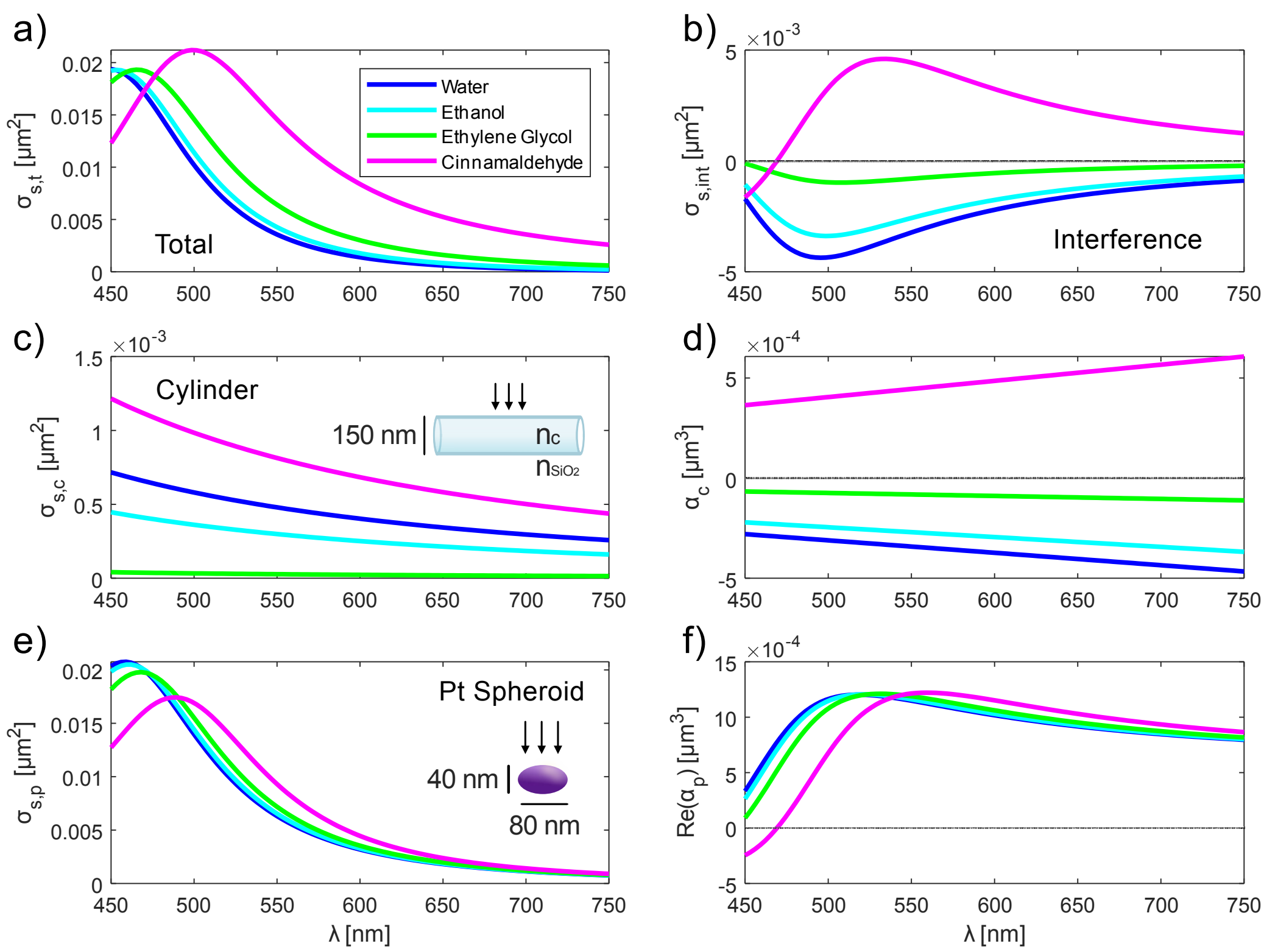

Figure S12. *Modeled spectra of cylinder and/or Pt spheroid systems with four different liquids in the cylinder/surrounding the particle. The cylinder is 150 nm in diameter and embedded in SiO_2, and the Pt metal spheroid is 40 nm high and 80 nm wide. Top-illumination of unpolarized light is assumed in all presented spectra. a) Total scattering cross section spectra of a Pt spheroid placed in cylinder filled with a liquid. b) interference term in the scattering cross section spectra of a Pt spheroid-cylinder system, i.e. the total scattering cross section spectrum subtracted by the cylinder and particle spectra. c) Cylinder scattering cross section spectrum. d) Polarizability for the cylinder. e) Pt spheroid scattering cross section. f) Real part of the Pt spheroid polarizability.*

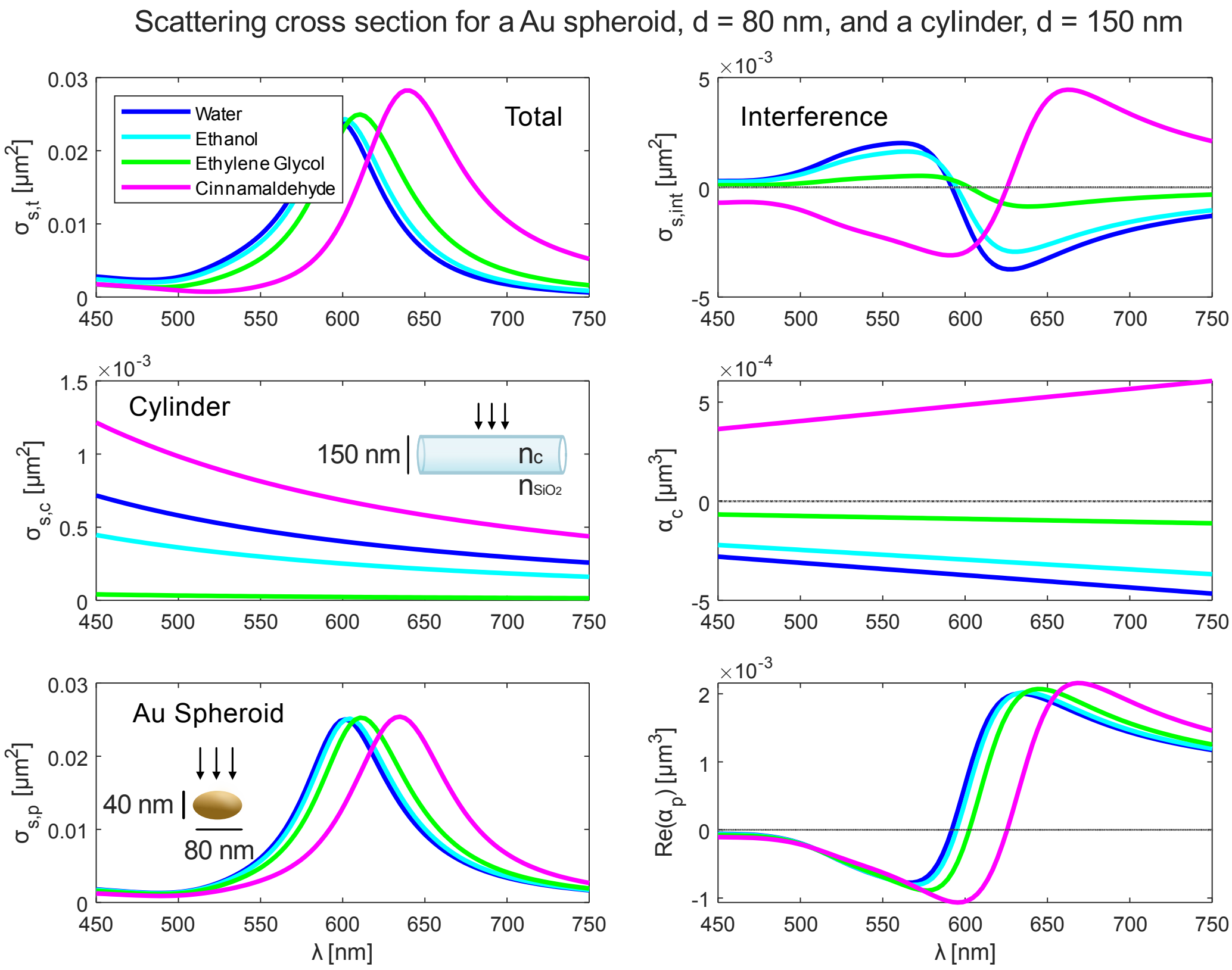

Figure S13. *Modeled spectra of cylinder and/or Au spheroid systems with four different liquids in the cylinder/surrounding the particle. The cylinder is 150 nm in diameter and embedded in SiO_2, and the Au metal spheroid is 40 nm high and 80 nm wide. Top-illumination of unpolarized light is assumed in all presented spectra. a) Total scattering cross section spectra of a Au spheroid placed in cylinder filled with a liquid. b) interference term in the scattering cross section spectra of a Au spheroid-cylinder system, i.e. the total scattering cross section spectrum subtracted by the cylinder and particle spectra. c) Cylinder scattering cross section spectrum. d) Polarizability for the cylinder. e) Au spheroid scattering cross section. f) Real part of the Au spheroid polarizability.*

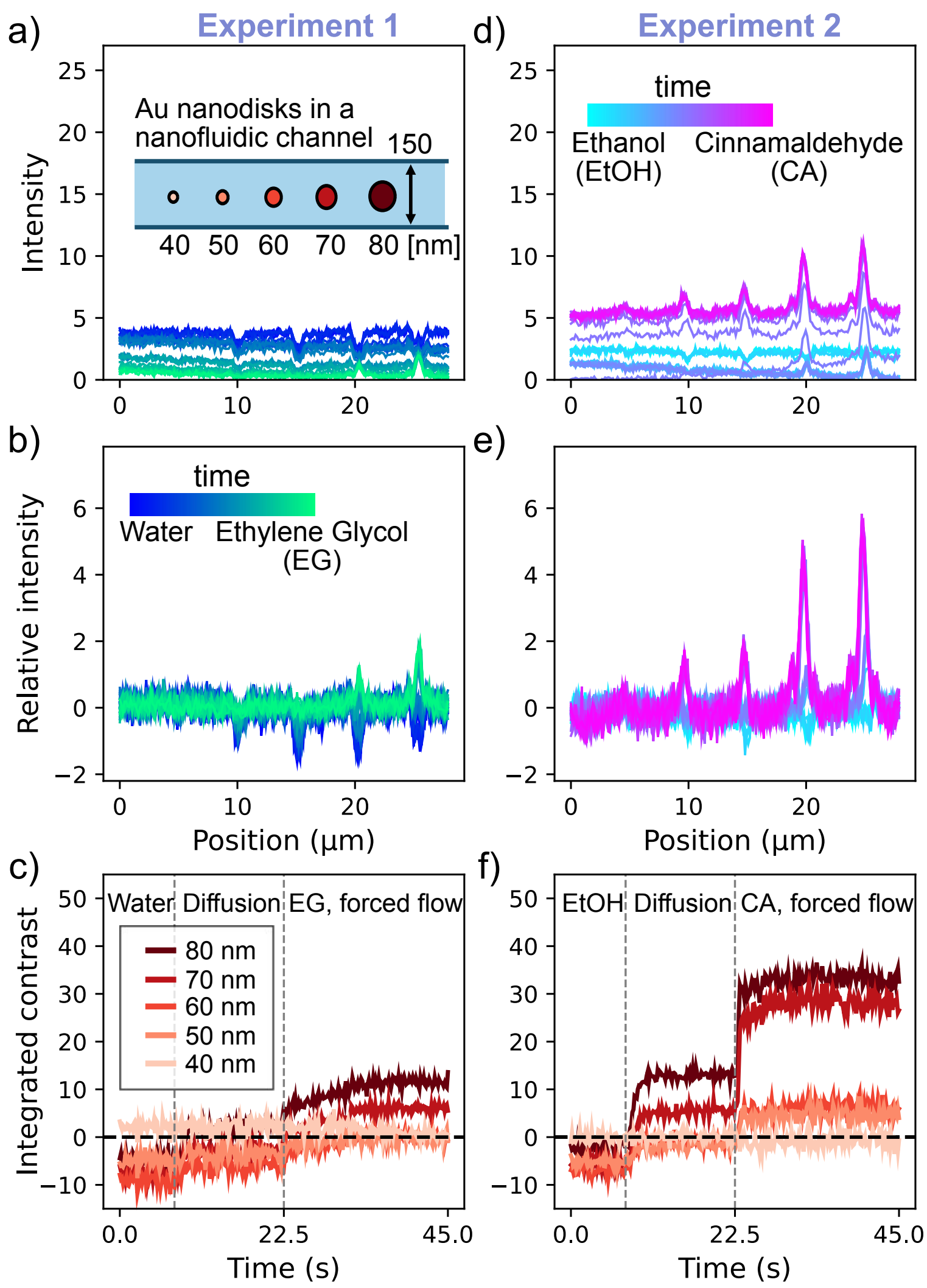

Figure S14. *In two experiments performed in a fully enclosed nanofluidic channel decorated with Au nanodisks the liquid in the channel was exchanged. The nanochannel is 150 nm wide and 70 nm deep and the nanodisks are ~40-~80 nm in diameter, have a deposition thickness of 40 nm, and are positioned 5 µm apart. a) In experiment 1 the liquid content of the nanofluidic channel is exchanged from water to ethylene glycol and imaged with a dark-field microscope over the course of 45 s. The graph displays the time evolution of the light scattering intensity along a nanochannel decorated with 5 Au nanodisks. The inset shows a schematic overview of the imaged channel with the nanodisk positions marked out. b) The scattering intensity of the five Au nanodisks are shown relative to the nanochannel intensity in experiment 1. c) Integrated scattering contrast of the intensity of the five Au nanodisks, obtained by summing the intensity relative to the nanochannel intensity, during experiment 1. The liquid state of the nanochannel is indicated by the dashed lines. d) shows the same system as in a) but imaged during experiment 2. In experiment 2 the liquid content of the nanofluidic channel is exchanged from ethanol to cinnamaldehyde. e) The relative scattering intensity of the five Au nanodisks in experiment 2. i) Integrated scattering contrast of the intensity of the five Au nanodisks relative to the nanochannel intensity during experiment 2.*

References

1. Bohren, C. F. & Huffman, D. R. *Absorption and Scattering of Light by Small Particles*. (John Wiley & Sons, 2008).
2. Malitson, I. H. Interspecimen Comparison of the Refractive Index of Fused Silica. *J. Opt. Soc. Am.* **55**, 1205–1209 (1965).
3. Kelly, K. L., Coronado, E., Zhao, L. L. & Schatz, G. C. The Optical Properties of Metal Nanoparticles: The Influence of Size, Shape, and Dielectric Environment. *J. Phys. Chem. B* **107**, 668–677 (2003).
4. Yu, R., Liz-Marzán, L. M. & García de Abajo, F. J. Universal analytical modeling of plasmonic nanoparticles. *Chem. Soc. Rev.* **46**, 6710–6724 (2017).
5. Johnson, P. B. & Christy, R. W. Optical Constants of the Noble Metals. *Phys. Rev. B* **6**, 4370–4379 (1972).
6. Werner, W. S. M., Glantschnig, K. & Ambrosch-Draxl, C. Optical Constants and Inelastic Electron-Scattering Data for 17 Elemental Metals. *J. Phys. Chem. Ref. Data* **38**, 1013–1092 (2009).
7. Langhammer, C., Yuan, Z., Zorić, I. & Kasemo, B. Plasmonic Properties of Supported Pt and Pd Nanostructures. *Nano Lett.* **6**, 833–838 (2006).
8. Langhammer, C., Kasemo, B. & Zorić, I. Absorption and scattering of light by Pt, Pd, Ag, and Au nanodisks: Absolute cross sections and branching ratios. *J. Chem. Phys.* **126**, (2007).
9. Altenburger, B., Fritzsche, J. & Langhammer, C. Visible Light Spectroscopy of Liquid Solutes from Femto- to Attoliter Volumes Inside a Single Nanofluidic Channel. *ACS Nano* **19**, 2857–2869 (2025).
